# Soluble Klotho protects against glomerular injury through regulation of ER stress response

**DOI:** 10.1038/s42003-023-04563-1

**Published:** 2023-02-22

**Authors:** Emmanuelle Charrin, Dina Dabaghie, Ilke Sen, David Unnersjö-Jess, Katja Möller-Hackbarth, Mikhail Burmakin, Rik Mencke, Sonia Zambrano, Jaakko Patrakka, Hannes Olauson

**Affiliations:** 1 KI/AZ Integrated Cardio Metabolic Center, Division of Pathology, Department of Laboratory Medicine, Karolinska Institutet at Karolinska University Hospital Huddinge, Stockholm, Sweden; 2 Division of Integrative Physiology, Department of Physiology and Pharmacology, Karolinska Institutet, Stockholm, Sweden; 3 Department II of Internal Medicine and Center for Molecular Medicine Cologne, University of Cologne, Faculty of Medicine and University Hospital Cologne, Cologne, Germany; 4 Royal Institute of Technology, Stockholm, Sweden; 5 Department of Medical Cell Biology, Uppsala University, Uppsala, Sweden; 6 Division of Pathology, Department of Pathology and Medical Biology, University of Groningen, University Medical Center Groningen, Groningen, The Netherlands; 7 Universitätsklinik für Neurochirurgie, Carl-von-Ossietzky Universität Oldenburg, Oldenburg, Germany; 8 Division of Renal Medicine, Department of Clinical Science, Intervention and Technology, Karolinska Institutet, Stockholm, Sweden

**Keywords:** Nephrons, Glomerular diseases

## Abstract

αKlotho (Klotho) has well established renoprotective effects; however, the molecular pathways mediating its glomerular protection remain incompletely understood. Recent studies have reported that Klotho is expressed in podocytes and protects glomeruli through auto- and paracrine effects. Here, we examined renal expression of Klotho in detail and explored its protective effects in podocyte-specific Klotho knockout mice, and by overexpressing human Klotho in podocytes and hepatocytes. We demonstrate that Klotho is not significantly expressed in podocytes, and transgenic mice with either a targeted deletion or overexpression of Klotho in podocytes lack a glomerular phenotype and have no altered susceptibility to glomerular injury. In contrast, mice with hepatocyte-specific overexpression of Klotho have high circulating levels of soluble Klotho, and when challenged with nephrotoxic serum have less albuminuria and less severe kidney injury compared to wildtype mice. RNA-seq analysis suggests an adaptive response to increased endoplasmic reticulum stress as a putative mechanism of action. To evaluate the clinical relevance of our findings, the results were validated in patients with diabetic nephropathy, and in precision cut kidney slices from human nephrectomies. Together, our data reveal that the glomeruloprotective effects of Klotho is mediated via endocrine actions, which increases its therapeutic potential for patients with glomerular diseases.

## Abstract (web summary)

Transgenic overexpression of αKlotho in hepatocytes results in protection against renal insults, possibly through modulation of the ER stress response by circulating αKlotho. In contrast, αKlotho overexpressed in podocytes is not renoprotective.

## Introduction

αKlotho (hereafter referred to as Klotho) is a type I membrane-bound protein mainly expressed in renal tubules, parathyroid cells, choroid plexus, and sinoatrial node^1–3^. Klotho knockout mice develop an accelerated aging phenotype characterized by hypervitaminosis D, phosphate retention, growth retardation, soft tissue calcification, infertility, osteoporosis, skin atrophy, and premature death^2^. The majority of these phenotypes are explained by membrane-bound Klotho forming complexes with fibroblast growth factor (FGF) receptor-1 that bind circulating FGF23 and regulate mineral metabolism^4^. However, cumulative evidence also points to independent functions of soluble Klotho, which is released from the cell membrane after enzymatic cleavage and can be detected in blood, urine, and cerebrospinal fluid^5^. Notably, renal Klotho levels are downregulated in patients with acute and chronic kidney disease^6^ as well as in numerous animal models of kidney injury and fibrosis^7–11^. Conversely, Klotho overexpression and treatment with recombinant protein have been demonstrated to be renoprotective in numerous animal models of kidney disease. Although the main effects appear to be on the tubulointerstitium, overexpression in a mouse model of glomerulonephritis alleviated both tubulointerstitial and glomerular damage^12^. Similar findings were reported in diabetic Ins2Akita mice, in which insertion of a Klotho transgene protected against glomerular injuries^13^. These two studies are supported by many other reports showing glomeruloprotective effects of soluble Klotho in several kidney disease models^9–11,14^. Considering the critical role of podocytes in the maintenance of glomerular integrity, it is biologically plausible that the beneficial actions of Klotho are mediated, at least partly, through effects on podocytes. In that respect, over the last 5 years, several studies have reported that Klotho is expressed in podocytes and inhibits proteinuria^15^, diabetes-induced oxidative stress^16–18^, podocyte apoptosis^16–19^, and glomerular hypertrophy^20^. Moreover, some single-cell sequencing studies have reported expression of Klotho in podocytes^21^, supporting a role for Klotho in podocyte biology. These results conflict with earlier studies reporting undetectable expression of Klotho in glomeruli^22–27^. As Klotho is a key player in renal physiology and disease, we set out to determine the expression and function of Klotho in murine and human podocytes in vitro, ex vivo, and in vivo. We hypothesized that Klotho is not expressed in podocytes and that its glomeruloprotective effects are mediated through systemic mechanisms rather than via local expression and/or direct effects on podocytes. To address this hypothesis, we assessed murine and human Klotho podocyte expression and generated mouse models with either podocyte-specific Klotho deletion or overexpression, and with systemically increased levels of soluble Klotho. The transgenic mice were challenged using nephrotoxic serum (NTS)-induced glomerulonephritis to examine the potentially protective effects of systemic or podocyte-derived Klotho on the glomerulus.

## Results

### Klotho is not significantly expressed by human or mouse podocytes

**Fig. 1:**
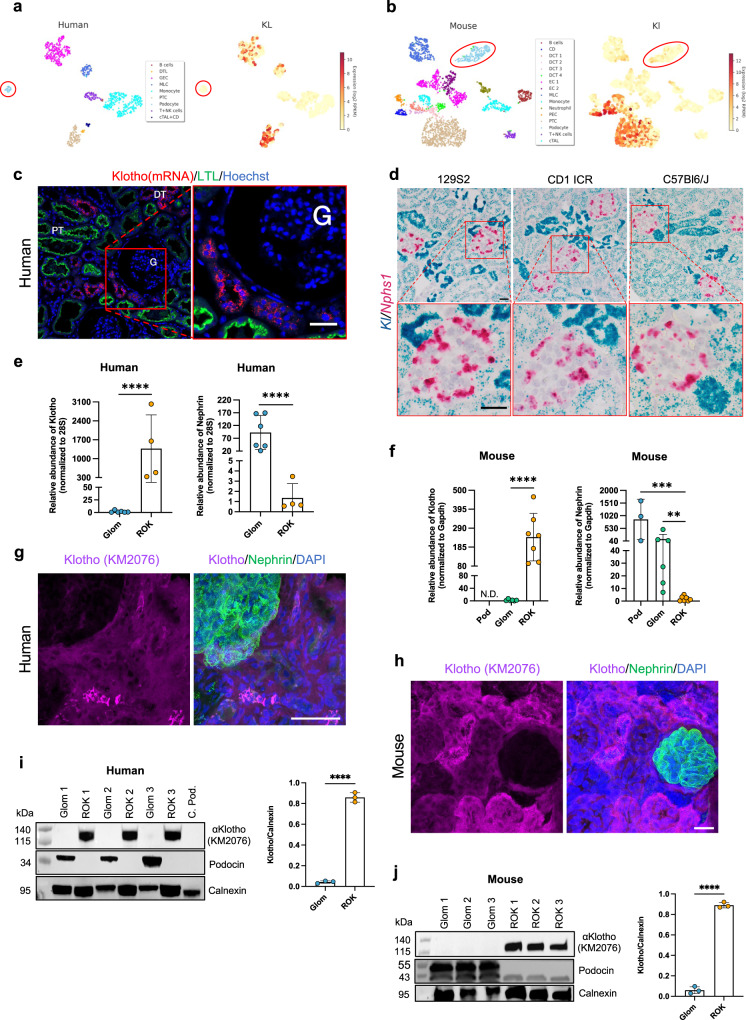
Expression of Klotho in normal human and mouse kidneys. **a**, **b** Human and mouse single-cell RNA-sequencing data extracted from Kidney glomerular single-cell atlas (data available at https://patrakkalab.se/kidney/). The left panels show a t-SNE plot of cell clusters based on the identification of highly expressed specific cell markers (left panels in supplementary Fig. 1). The t-SNE plot (right panels) shows that Klotho expression in the different clusters. The podocytes circled in red. DTL: descending thin limb of Henle’s loop; GEC: glomerular endothelial cells; MLC: Mesangial-like cells; PTC: Proximal tubular cells; T + NK: T + natural killer lymphocytes; cTAL + CD: cortical thick ascending limb of Henle’s loop + collecting duct. CD: collecting ducts; DCT: distal convoluted tubules; EC: endothelial cells; PEC: glomerular parietal epithelial cells; cTAL: cortical thick ascending limb of Henle’s loop. **c** In situ hybridization of human kidney cortex where Klotho (red) was co-labeled with Lotus tetragonolobus lectin (LTL, green) and Hoechst (blue). **d** In situ hybridization of mouse kidney cortex from 3 different strains (129S2, CD1 ICR, C57BL6/J) where Klotho (blue) and Nephrin (pink) were labeled. **e**, **f** RT-qPCR for Klotho in rest of kidney (ROK), glomerular (glom) fraction and in (**f**) FAC sorted mouse podocytes (Pod). 28S (**e**) and GAPDH (**f**) genes were used as a housekeeping gene and nephrin to validate the purity of glomerular fraction and isolated podocytes. Values are expressed as mean ± SD (*n* = 3). **p < 0.01; ***p < 0.001; ****p < 0.0001. **g**, **h** Dual labeling with a podocyte marker nephrin (green) and Klotho (purple) in both human (**g**) and mouse (**h**). **i**, **j** Western blotting in the rest of kidney (ROK) and glomeruli fraction (Glom). Calnexin was used as a loading control and podocin antibody validates the purity of isolated glomeruli. Data are expressed as mean ± SD (*n* = 3). ****p < 0.0001. Scale bars: 100 μm (**c**); 20 μm (**d**), 50 μm (**g**), 20 μm (**h**). Statistical significance between groups was determined using unpaired t-test; more than 2 groups were compared with one-way ANOVA.

We analyzed the expression of Klotho in human and mouse kidney using single-cell RNA sequencing, in situ hybridization, qPCR, Western blotting, and immunofluorescence staining. Exploring our recently published single-cell RNA-sequencing data^28^, the highest expression of Klotho was found in distal tubular cells, and lower expression was seen in proximal tubular cells. No or extremely low expression was observed in human (Fig. 1a) and mouse (Fig. 1b) podocytes. The absence of cross contamination between the different cell clusters was confirmed using well-known markers for each specific cell type (i.e., Nphs1 for podocytes; *Cdh1* for distal tubules; *Slc34a1* for proximal tubules; supplementary Fig. 1). In situ hybridization showed strong Klotho expression in distal tubuli and lower expression in proximal tubuli in sections from human kidney and in kidney sections from the three different mouse strains (129S2, CD1 ICR, and C57BL6) tested (Fig. 1c, d). In contrast, no signal, or only scattered single dots were detected in glomerular cells. In line with this, qPCR of isolated human glomeruli showed Klotho expression of 0,1% compared to the rest of kidney fraction (Fig. 1e). Similarly, transcript level of Klotho was drastically lower in mouse glomeruli compared to the rest of kidney and was below the detection threshold in FAC-sorted podocytes (Fig. 1f). Protein expression studies using two different antibodies in both formalin-fixed paraffin-embedded (FFPE) and frozen sections supported the gene expression data of Klotho in different renal cell types (Supplementary Figs. 2 and 3). In addition, in super-resolution STED images of both human and mouse kidney, Klotho expression was seen in distal and proximal tubuli, but was not found in the glomeruli (Fig. 1g, h). This was confirmed by Western blotting demonstrating that Klotho was abundantly expressed in the tubulointerstitial fraction but was absent in glomerular lysates from human and mouse kidney (Fig. 1i, j). Taken together, our data strongly suggest that Klotho is not expressed by human or mouse podocytes, or by any other glomerular cell type. However, we cannot completely rule out a low-grade expression of Klotho in podocytes.

### Deletion of the Klotho gene in podocytes does not result in a glomerular phenotype or aggravate outcome after glomerular injury

**Fig. 2:**
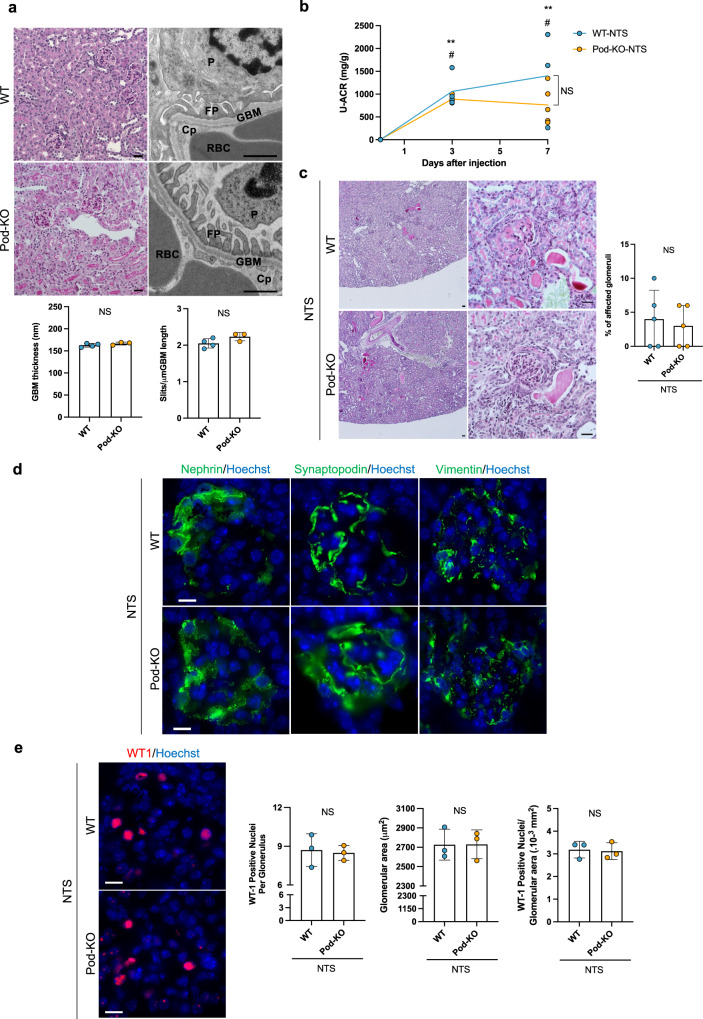
Characterization of Pod-KO at baseline and after NTS-induced injury. **a** Representative images of PAS staining (left panels) from 5-month-old Pod KO mice. Transmission electron microscopy examination (right panels) and quantification of mean glomerular basement membrane (GBM) thickness and the number of slits per μm GBM in WT and Pod-KO mice. Cp: capillary lumen, FP: foot process, GBM: glomerular basement membrane, P: podocyte, RBC: red blood cell. NS: not significant. **b** Urinary albumin/creatinine ratios (U-ACR) in WT and Pod-KO mice, 0 day (*n* = 4 WT; *n* = 5 Pod-KO), 3 days (*n* = 4 WT; *n* = 3 Pod-KO), and 7 days (*n* = 3 WT; *n* = 5 Pod-KO) after injection of NTS. **p < 0.01 WT day 0; ^##^*p* < 0.01 vs^.^ Pod-KO day 0. NS: Not significant. **c** PAS staining in WT and Pod-KO glomeruli 7 days after the induction of disease (*n* = 5). **d** Immunofluorescent stainings of podocyte-specific markers nephrin, synaptopodin, and vimentin in both WT and Pod-KO after NTS injection. **e** Immunofluorescent staining of WT1. The number of WT-1 positive podocytes (left panel) in glomeruli as well as glomerular area (middle panel) and WT-1 positive podocytes/glomerular area were plotted in WT and Pod-KO after NTS injection. WT-1 positive cells were counted in 20 randomly chosen glomeruli. NS: not significant. Scale bars: 30 μm (PAS), 1 μm (tEM) (**a**); 50 μm, 30 μm (**c**); 10 μm (**d**, **e**). Statistical significance between groups was determined using unpaired t-test or mixed-effects analysis followed by multiple comparisons.

As Klotho expressed in podocytes has been reported to protect against podocyte injury^15–20^, and targeted deletion of Klotho has been shown to have potential effects even in cell types with close to unmeasurable levels of Klotho expression^22,29–31^, we generated mice with a podocyte-specific deletion of the Klotho gene (Pod-KO) (Supplementary Fig. 4). Since we could not detect Klotho expression in podocytes from wild-type mice, we validated this approach using in situ hybridization for Klotho in kidneys from mice with systemic (β-actin-KO) or proximal tubule-specific (Pepck-KO) deletion of Klotho (Supplementary Fig. 4c), as previously reported^22,32^. The activity of Cre recombinase in podocytes was validated by crossing the Podocin-cre line with ^Gt(ROSA)26Sortm14(CAG-tdTomato)^Hze/J mouse (Supplementary Fig. 4e). Pod-KO mice were viable, fertile, and developed normally. Renal histology and expression of podocyte proteins were normal in 20-week-old Pod-KO mice as demonstrated by light microscopy and immunofluorescence stainings (Fig. 2a and Supplementary Fig. 5). Electron microscopic examination revealed intact ultrastructure of the filtration barrier in Pod-KO mice, including normal interdigitating podocyte foot processes and slit diaphragms (Fig. 2a). Consistently, no albuminuria—the most common readout of impaired podocyte function—was observed in urine samples from Pod-KO mice (Supplementary Fig. 4f). The data indicate that the deletion of Klotho in mouse podocytes does not result in any obvious alterations of the structure or function of the glomerulus.

Next, we examined whether deletion of Klotho in podocytes may alter their function during disease processes. We induced glomerulonephritis in Pod-KO mice with a single i.v. injection of nephrotoxic serum (NTS), as previously described^33,34^. As expected, all mice developed albuminuria at 3 and 7 days after injection of NTS (Fig. 2b). However, albuminuria levels did not differ between WT and Pod-KO mice. Moreover, histopathological changes were similar in both groups as shown by semi-quantitative scoring (Fig. 2c). Podocyte differentiation markers nephrin, synaptopodin, and vimentin were similarly affected in WT and Pod-KO glomeruli exposed to NTS (Fig. 2d). Furthermore, podocyte loss and glomerular hypertrophy were similar in both groups after NTS-induced glomerulonephritis, as showed by the lack of difference in WT1-positive nuclei and glomerular area (Fig. 2e).

### Transgenic mice overexpressing full-length human Klotho in hepatocytes or podocytes display a normal gross phenotype and unchanged mineral metabolism

**Fig. 3:**
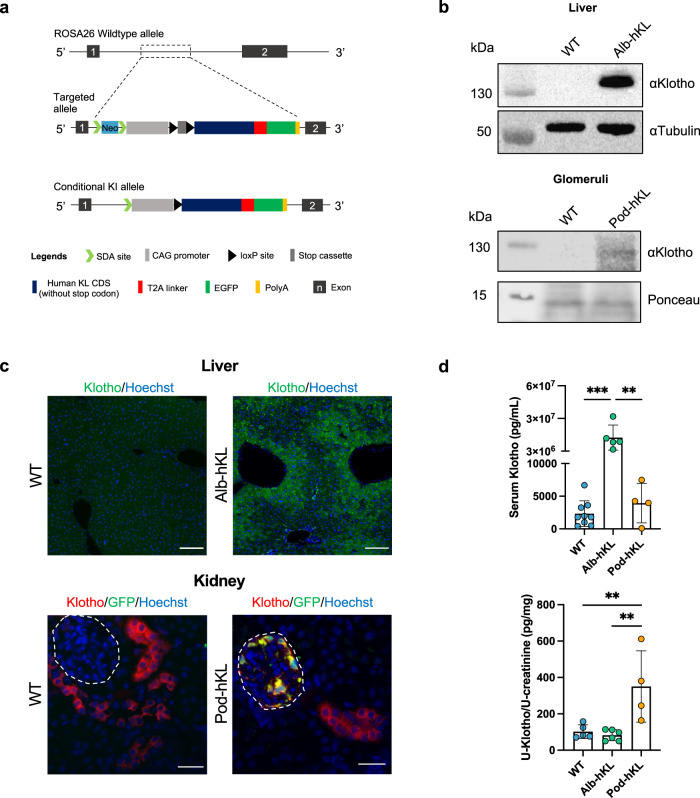
Generation of conditional full-length human Klotho knock-in allele and targeted insertion of human Klotho in Alb-hKL and Pod-hKL mice. **a** Schematic representation of ROSA26 wild-type allele (top), targeted allele (middle), and conditional knock-in allele (bottom). In the latter, full-length human Klotho expression is specifically induced in hepatocytes or podocytes by using Cre–LoxP recombination system. Albumin- or podocin-cre mice were crossed with mice expressing the targeting vector, containing a lox-STOP-lox cassette between the splice acceptor sites and Klotho cDNA under the *CAG* promoter, that was introduced into the ROSA26 locus. **b** Western blotting of whole liver extracts from WT and Alb-hKL (top) and glomeruli-enriched lysates from WT and Pod-hKL mice (bottom). **c** Representative immunofluorescent stainings of Klotho and GFP in liver (top) and kidney (bottom) of Alb-hKL and Pod-hKL, respectively. **d** Serum (top) and urine (bottom) Klotho levels in WT (*n* = 9), Alb-hKL (*n* = 5), and Pod-hKL (*n* = 4) mice. **p < 0.01; ***p < 0.001. Scale bars: 100 μm, 30 μm (**c**). Statistical significance between groups was determined using one-way ANOVA followed by multiple comparisons.

To understand whether the reported glomeruloprotective effects of Klotho are mediated via local or systemic effects, we generated a transgenic mouse lines in which human full-length Klotho were overexpressed in either podocytes or hepatocytes. In these transgenic mice, a cassette containing a lox-PolyA-lox construct next to the full-length human KL (hKL) gene fused to a T2A linker-EGFP, under the CAG promoter was introduced into the Rosa26 locus (Fig. 3a). The floxed hKL mice were crossed with mice expressing cre recombinase under either an albumin or a podocin promoter and the heterozygous offspring (Alb-hKL and Pod-hKL) and wild-type littermates (WT) were used in age-matched groups for the study. To confirm a successful insertion of the transgene, we analyzed Klotho protein expression using Western Blotting and immunofluorescence in these mice (Fig. 3b, c). Indeed, Klotho protein was found in Alb-hKL liver lysate and Pod-hKL glomerular fraction but not in WT liver lysate or WT glomerular fraction (Fig. 3b). GFP was observed in Alb-hKL hepatocytes and in Pod-hKL podocytes while this signal was absent in WT littermates (Fig. 3c). Alb-hKL and Pod-hKL mice were viable, fertile and did not differ in size or display any gross phenotypical or behavioral abnormalities (Table 1). Of note, hepatic expression of β-Klotho was not different between WT and Alb-hKL mice (Supplementary Fig. 6c). Notably, serum Klotho levels were increased by more than 6000-fold on average in Alb-hKL compared to WT and Pod-hKL mice (Fig. 3d) while serum Fgf23 was approximately two-fold higher (Table 1). Despite the extreme elevation in serum Klotho, levels of urinary Klotho (Table 1) and in whole kidney lysates (Supplementary Fig. 6b) were not higher in Alb-hKL compared to WT mice. Serum phosphate and calcium, and fractional excretion of phosphate and calcium did not differ between genotypes. Of note, urinary Klotho was significantly higher in Pod-hKL compared to WT and Alb-hKL (Fig. 3d), whereas serum Klotho was similar in Pod-hKL and WT mice.

**Table 1:** General characteristics, serum, and urinary biochemistry in WT, Alb-hKL, and Pod-hKL mice at baseline.

	WT	Alb-hKL	Pod-hKL
Age—Female (weeks)	10.7 ± 2.2	11.1 ± 1.7	12.2 ± 3.5
Age—Male (weeks)	10.4 ± 1.3	9.9 ± 0.9	9.8 ± 1.6
Weight—Female (g)	21.2 ± 2.1	19.9 ± 1.9	26.1 ± 4.4
Weight—Male (g)	29.0 ± 2.9	26.6 ± 2.2	27.0 ± 2.2
Female/male	12/12	11/9	5/5
Serum	*n* = 14	*n* = 10	*n* = 4
Creatinine (mg/dL)	0.153 ± 0.04	0.183 ± 0.04	0.113 ± 0.03
Calcium (mg/dL)	9.49 ± 2.46	10.64 ± 2.47	10.64 ± 1.18
Phosphate (mg/dL)	10.51 ± 1.67	11.60 ± 1.07	10.73 ± 2.61
Intact Fgf23 (pg/mL)	139.3 ± 64.8	306.1 ± 189.1#	116.4 ± 19.2
Urine	*n* = 14	*n* = 10	*n* = 4
FECa (%)	0.43 ± 0.24	1.03 ± 0.83	0.44 ± 0.40
FEPi (%)	0.53 ± 0.49	0.39 ± 0.49	0.77 ± 0.04

*FECa* fractional excretion of calcium, *FEPi* fractional excretion of phosphate.

#*p* < 0.05 vs. WT.

### Higher systemic Klotho protects the glomeruli against injury

**Fig. 4:**
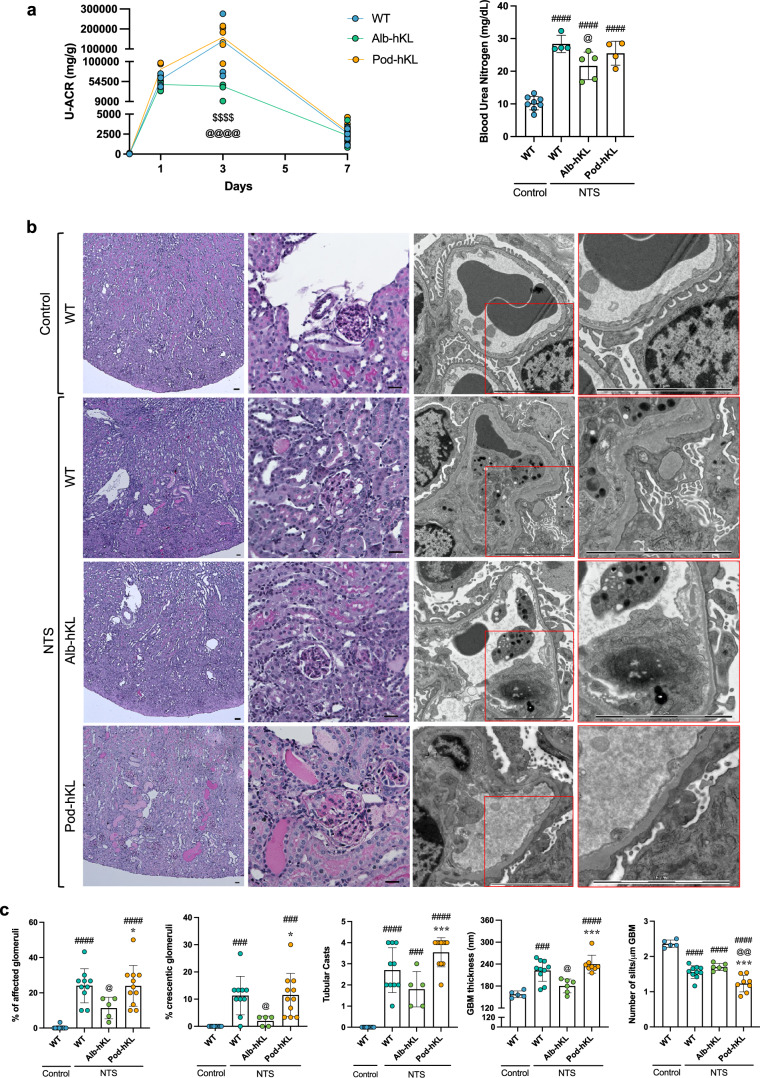

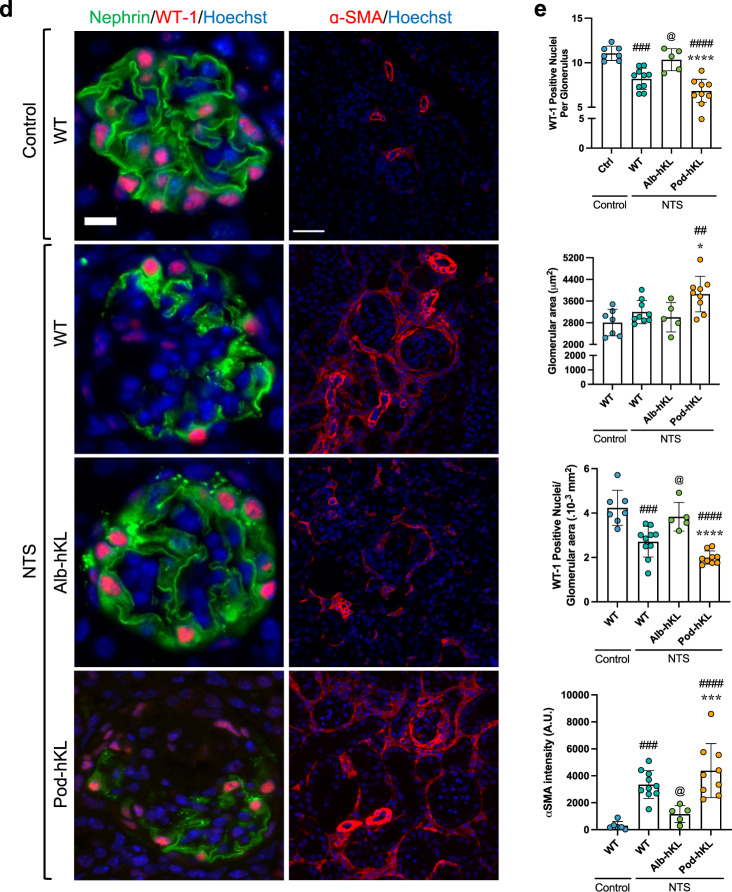
Hepatocyte-specific overexpression of Klotho ameliorates renal injury and proteinuria in a mouse model of NTS-induced glomerulonephritis. **a** Left panel: Urinary albumin/creatinine ratio (U-ACR) in WT, Alb-hKL, and Pod-hKL, 0, 1, 3, and 7 days after injection of NTS. ^$$$$^*p* < 0.0001 vs. Pod-hKL and ^@@@@^*p* < 0.0001 vs. WT. Right panel: Blood urea nitrogen in the different groups without injury (control mice) or 7 days after injection of NTS (NTS mice). ^####^*p* < 0.0001 vs. WT-control; ^@^*p* < 0.05 vs. WT-NTS. **b** Representative images of PAS (left panels) and TEM (right panels) of kidney cortex from WT-control and WT, Alb-hKL, Pod-hKL 7 days after NTS injection. **c** Percentage of affected and crescentic glomeruli were quantified from 30 randomly selected glomeruli in the corresponding PAS staining. Tubular casts were assessed over the whole cross-section giving a score between 0, no cast to 5, >80% of the tubules are affected (*n* = 11 WT-control; *n* = 10 WT-NTS; *n* = 5 Alb-hKL-NTS; *n* = 11 Pod-hKL-NTS). Quantification of mean glomerular basement membrane (GBM) thickness and the number of slits per μm GBM (*n* = 5 WT-control; *n* = 10 WT-NTS; *n* = 6 Alb-hKL-NTS; *n* = 8 Pod-hKL-NTS). ^###^*p* < 0.001, ^####^*p* < 0.0001 vs. WT-control; ^@^*p* < 0.05, ^@@^*p* < 0.01 vs. WT-NTS and *p < 0.05, ***p < 0.001 vs. Alb-hKL-NTS. **d** Representative images of immunofluorescent stainings of Nephrin, WT-1, and α-SMA in the different groups. **e** Quantification of the number of WT-1 positive podocytes in glomeruli (left panel), glomerular area (middle panel), and WT-1 positive podocytes/glomerular area (right panel) (*n* = 7 WT-control; *n* = 9 WT-NTS; *n* = 5 Alb-hKL-NTS; *n* = 9 Pod-hKL-NTS). α-SMA positive area was counted per cortical cross section (10 sections per sample; *n* = 6 WT-control; *n* = 10 WT-NTS; *n* = 5 Alb-hKL-NTS; *n* = 9 Pod-hKL-NTS). ^##^*p* < 0.01, ^###^*p* < 0.001, ^####^*p* < 0.0001 vs. WT-control; ^@^*p* < 0.05 vs. WT vs. WT-NTS and *p < 0.05, ***p < 0.001, ****p < 0.0001 vs. Alb-hKL-NTS. Scale bars: 50 μm, 30 μm, 5 μm (**b**), 10 μm, 50 μm (**d**). Statistical significance between groups was determined using one-way or two-way repeated measures ANOVA followed by multiple comparisons.

To test whether increased expression of Klotho in hepatocytes or in podocytes protect against glomerular injury, we induced NTS glomerulonephritis in Alb-hKL and Pod-hKL mice. All mice developed albuminuria that peaked on day 3 and returned closer to baseline levels on day 7 (Fig. 4a). However, the peak in uACR was significantly higher (about 3-fold) in WT and Pod-hKL at day 3 compared to Alb-hKL. Similarly, BUN was lower in Alb-hKL compared to WT and Pod-hKL 7 days after the single injection of NTS (Fig. 4a). Of note, serum creatinine did not differ between the mouse lines at seven days post NTS-induced kidney injury (Table 2), potentially due to its low sensitivity as a marker of renal injury, and/or because most of the acute injury was resolved at this timepoint. Histological examination revealed a substantial amelioration in glomerular injury in Alb-hKL mice (Fig. 4b) with a lower number of affected glomeruli, crescent formation, and a reduced GBM thickness (Fig. 4c). While the number of nuclei positive for the podocyte marker WT1 was significantly lower in WT and Pod-hKL mice after NTS-induced injury compared to control mice, the number of WT1-positive cells remained unchanged in Alb-hKL mice after NTS injury (Fig. 4d, e). Finally, there was markedly less interstitial ɑSMA-positivity in Alb-hKL kidneys compared to WT and Pod-hKL kidneys (Fig. 4d, e). These data illustrate that systemic overexpression of Klotho in Alb-hKL mice helps to preserve the renal structure and function in response to glomerular injury, without affecting serum phosphate or calcium concentrations (Table 2). In contrast, overexpression of Klotho in podocytes did not confer any protection against glomerular injury or interstitial fibrosis.

**Table 2:** Serum and urinary biochemistry in WT, Alb-hKL, and Pod-hKL mice 7 days after NTS injection compared to WT-control.

	Control	NTS		
	WT, *n* = 14	WT, *n* = 16	Alb-hKL, *n* = 12	Pod-hKL, *n* = 9
Serum				
Creatinine (mg/dL)	0.153 ± 0.04	0.228 ± 0.02^####^	0.210 ± 0.02^##^	0.231 ± 0.06^###^
Calcium (mg/dL)	9.49 ± 2.46	6.92 ± 1.21^###^	6.63 ± 1.08^###^	7.86 ± 0.66
Phosphate (mg/dL)	10.51 ± 1.67	9.49 ± 1.83	9.64 ± 1.36	8.29 ± 0.97^#^
Klotho (ng/mL)	2.34 ± 1.97****	1.78 ± 1.15****	1.3.10^4^ ± 6.9.10^3^	8.71 ± 4.69****
Intact Fgf23 (pg/mL)	153.9 ± 55.5	290.5 ± 102.5^#^	426.8 ± 146.5^####^	507.5 ± 239.8 ^####,@@^
Urine				
FECa (%)	0.43 ± 0.24	0.90 ± 0.59	1.26 ± 0.97	1.27 ± 1.03
FEPi (%)	0.53 ± 0.49	0.24 ± 0.33	0.27 ± 0.37	0.12 ± 0.18
U-Klotho/U-Creatinine (pg/mg)	102.1 ± 37.7	95.1 ± 13.1	177.0 ± 152.9	102.1 ± 33.5

*FECa* fractional excretion of calcium, *FEPi* fractional excretion of phosphate.

^#^*p* < 0.05.

^##^*p* < 0.01.

^###^*p* < 0.001.

^####^*p* < 0.0001 vs. Control WT.

^@@^*p* < 0.01 vs. NTS WT.

****p < 0.0001 vs NTS Alb-hKL.

### The glomeruloprotective effects of soluble Klotho involve regulation of the endoplasmic reticulum stress/unfolded protein response pathways

**Fig. 5:**
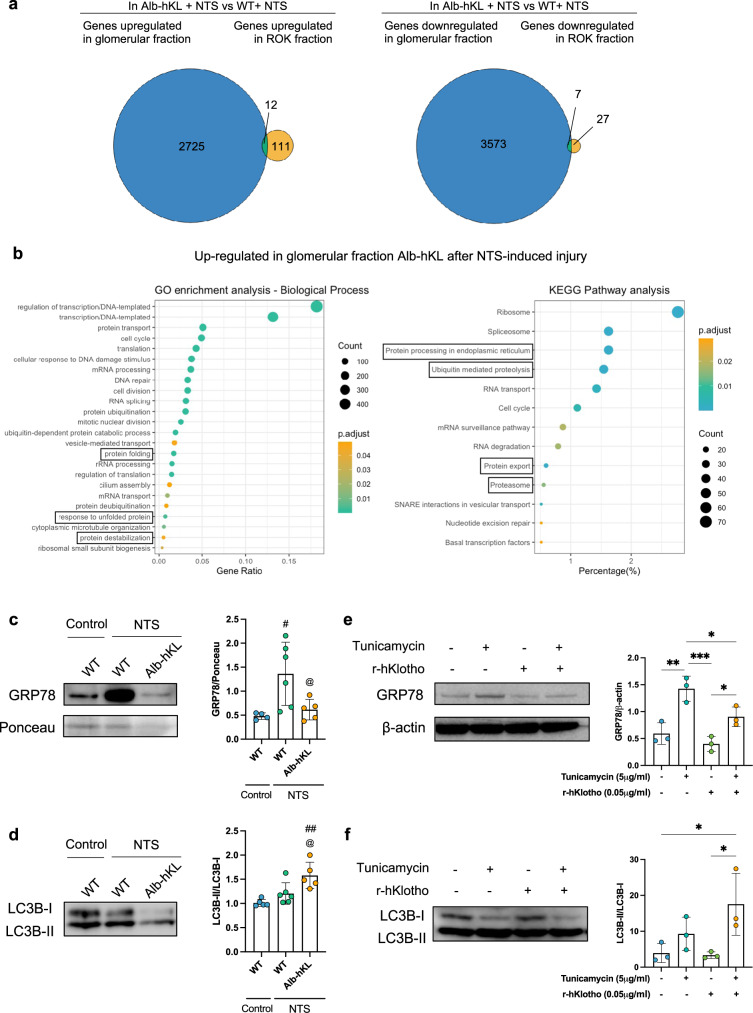
Circulating Klotho mediates its effects through the regulation of ER stress/UPR pathways in response to NTS-induced injury. **a** Venn diagrams illustrate the number of genes significantly regulated by Klotho overexpression in glomeruli-enriched or tubuli-enriched (ROK) fractions as well as their overlap in Alb-hKL compared to WT animals. **b** Functional enrichment analyses (GO-term analysis on the left panel; KEGG pathway analysis on the right panel) conducted on the upregulated genes in the glomerular fraction shown in (**a**). Only significant enrichments are shown (DAVID scores ≥1.3). Black boxes highlight the GO-term and KEGG pathways which we focused on in further analyses. **c**, **d** Representative western blotting (left panels) of GRP78/BiP (**c**) and LC3B (**d**) in glomeruli-enriched lysates from the different groups. 20 μg of proteins per well were loaded. Densitometry quantification (right panels) for GRP78/BiP and LC3B (*n* = 5 WT-control; *n* = 6 WT-NTS; *n* = 5 Alb-hKL-NTS) was performed. ^#^*p* < 0.05, ^##^*p* < 0.01 vs^.^ WT-control; ^@^*p* < 0.05 vs. WT-NTS. **e**, **f** Representative western blotting (left panels) of GRP78/BiP (**e**) and LC3B (**f**) in immortalized mouse podocytes after treatment with tunicamycin (5 μg/ml) and/or recombinant human Klotho (r-hKlotho, 0.05 μg/ml) for 16 h. 10 μg of proteins per well were loaded. Densitometry quantification (right panels) was performed on 3 replicates for each condition. *p < 0.05; **p < 0.01; ***p < 0.001. Statistical significance between groups was determined using one-way ANOVA.

Renal protection by soluble Klotho have been associated to several pathways including TGF-ß^35^, IGF-1^36^, Wnt^37^, TRPC6^15^, Nrf2^16^, and VEGF^38^. However, its putative receptors have not been definitely identified. Herein, we employed an unbiased approach to elucidate the mechanism by which soluble Klotho exerts protection of the glomeruli. We performed RNA sequencing of glomeruli- and tubulointerstitial fractions of WT and Alb-hKL mice 7 days after injection of NTS. From the generated datasets, 2725 upregulated and 3573 downregulated genes were identified in the glomerular fraction of Alb-hKL mice compared to WT controls, while only 111 upregulated and 27 downregulated genes were found in the tubulointerstitial fraction (Fig. 5a). Thus, despite observing protective effects on both compartments, most gene expression changes are found in the glomerular fraction in Alb-hKL mice after NTS-induced glomerulonephritis. Next, using Gene Ontology (GO)-term enrichment and the Kyoto Encyclopedia of Genes and Genomes (KEGG) pathway analyses, we found that significantly upregulated genes in the glomerular fraction of Alb-hKL were enriched for genes related to protein homeostasis and response to ER stress (e.g., protein ubiquitination, protein folding, response to unfolded protein, protein processing in endoplasmic reticulum) compared to WT mice (Fig. 5b). To verify that our mRNA data translate to the protein level, we ran Western blotting on glomerular lysates from WT and Alb-hKL after NTS glomerulonephritis (Fig. 5c, d). We observed that GRP78, a master regulator of the unfolded protein response (UPR) pathway^39–41^ was higher after injection of NTS, validating induction of ER stress in our model. Importantly, GRP78 was markedly lower in Alb-hKL compared to WT mice (Fig. 5c). Autophagy is another mechanism for the clearance of components like misfolded proteins and dysfunctional organelles to maintain protein homeostasis^42^. The conversion of LC3B-I to LC3B-II was higher in the glomerular fraction from Alb-hKL mice, suggesting higher autophagosome formation compared to in WT mice (Fig. 5d). To validate our in vivo findings mechanistically, we treated cultured mouse podocytes with tunicamycin, a well-established chemical inducer of ER stress (Fig. 5e, f). While tunicamycin tripled the expression of GRP78, addition of recombinant human Klotho in the cell culture medium significantly reduced GRP78 levels (Fig. 5e) and elevated LC3B-II conversion (Fig. 5f).

### Klotho expression associates to ER stress in patients with diabetic nephropathy and recombinant Klotho regulates ER stress in human kidney tissue ex vivo

**Fig. 6:**
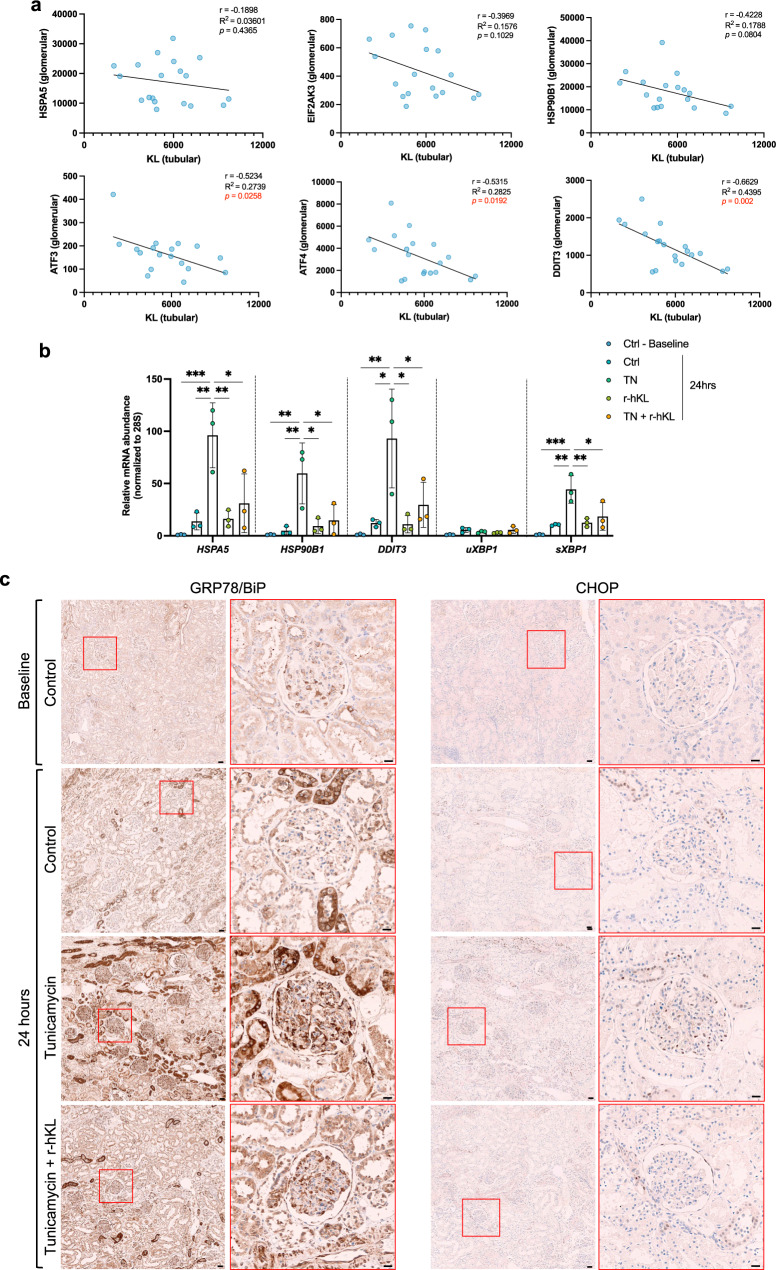
Low mRNA levels of Klotho is associated with high mRNA levels of ER stress markers in diabetic nephropathy patients and recombinant human Klotho regulates ER stress markers expression in response to Tunicamycin in an ex vivo model of human precision cut kidney slices. **a** Correlation between mRNA level of Klotho (KL) in tubular fraction and mRNA level of ER stress markers in glomerular fraction i.e., HSPA5 coding for GRP78/BiP, HSP90B1 coding for GRP94, EIF2AK3 coding for PERK, ATF4, ATF3, DDIT3 coding for CHOP. Data are extracted from Levin et al.^48^. **b** Using RT-qPCR, mRNA expression of HSPA5, HSP90B1, DDIT3, unspliced and spliced XBP1 was measured in 300-μm-thick human kidney slices directly after slicing procedure (Baseline) or after a 24 h-treatment with either tunicamycin (TN, 1 μM) or recombinant human Klotho (r-hKL, 0.05 μg/ml). 28S was used as a housekeeping gene. Data represent the mean ± SD of 1 independent experiment performed in triplicates. *p < 0.05; **p < 0.01; ***p < 0.001. **c** Representative images of immunoperoxidase stainings for GRP78/BiP (left panel) and CHOP (right panel) in human kidney slices. Statistical significance between groups was determined using Pearson’s correlation test or one-way ANOVA followed by multiple comparisons.

Maladaptive ER stress response has been linked to the development and progression of diabetic nephropathy (DN)^43–46^. Furthermore, renal Klotho expression has been reported to be lower in patients with DN compared to other types of CKD^47^. We therefore analyzed the correlation between tubular Klotho and glomerular ER stress/UPR markers in kidney biopsies from DN patients^48^ (Fig. 6a). Higher tubular Klotho transcript levels were associated with lower transcript levels of the glomerular ER stress/UPR markers (ATF4, ATF3, DDIT3), downstream of PERK, one of the three major arms of the UPR. To confirm the direct action of Klotho on ER stress in human kidneys, we used precision cut kidney slices (PCKS)^49,50^ obtained from tumor-free part of renal cancer nephrectomies. Treatment with tunicamycin caused an increase in *HSPA5*, *HSP90B1*, *DDIT3*, and *sXBP1* expression ranging from 50- to 100-fold compared to control conditions (Fig. 6b). Addition of recombinant human Klotho to the medium significantly lowered the expression of these markers. The effects of recombinant Klotho on GRP78 (encoded by the *HSPA5* gene) and CHOP (encoded by the *DDIT3* gene) were confirmed by immunohistochemistry (Fig. 6c).

## Discussion

Podocyte dysfunction is a well-established mechanism behind the progression of proteinuria and kidney diseases. Accumulating evidence strongly supports that Klotho has a protective role against various forms of glomerular injury. Although many pathways through which these protective effects are mediated have been proposed, the mechanism(s) of action remain unclear. Importantly, there are conflicting data on whether Klotho is expressed in podocytes or not. In this study, we addressed these topics through various approaches. First, we analyzed the expression of Klotho at transcript and protein levels in human and mouse kidneys. Using several different methods, we were unable to detect Klotho in human or mouse podocytes, at baseline or after injury. Second, to rule out potential effects of extremely low Klotho expression in podocytes, we generated podocyte-specific Klotho knockout mice and demonstrate that complete deletion of Klotho from podocytes does not induce a glomerular phenotype, nor does it affect the outcome after glomerular injury. Third, we generated a transgenic mouse line allowing conditional overexpression of human full-length Klotho. Notably, mice overexpressing Klotho in podocytes were not protected against glomerular injury. However, increased circulating concentrations of soluble Klotho derived from hepatocytes protected against glomerular injury and helped to maintain glomerular integrity and function. Functional studies in mouse and human tissue identified modulation of ER stress/UPR pathways as a potential mechanism of action.

At its discovery in 1997^2^, renal Klotho expression was described in cortical tubules, with no expression in glomerular cells. This expression pattern was later confirmed by numerous studies in various species, on transcript as well as on protein level^51^. However, more recently several studies have reported Klotho expression also in the glomeruli, most frequently in podocytes^15–20^. Furthermore, Klotho has been reported to protect the podocytes from injury through various mechanisms. Expression of Klotho in podocytes is of importance to understand whether its effects on podocytes are direct or indirect, as it is a large protein (approximately 130 kDa) unlikely to pass the glomerular filtration barrier. Hence, soluble Klotho released from extraglomerular cells would not be able to reach the podocytes and exert its protective effects directly. Herein we wanted to elucidate (i) whether podocytes express Klotho, and (ii) how the reported glomeruloprotective effects of Klotho are mediated.

Using two previously validated antibodies and in situ hybridization we failed to detect Klotho expression in podocytes in FFPE or frozen sections from mouse and human kidneys. Similarly, we could not detect Klotho in isolated podocytes by qPCR or Western blot. The specificity of the methods was confirmed using kidneys from tissue-specific and global Klotho knockout mice. Our results from these experiments indicate that Klotho is absent or expressed only at extremely low levels in podocytes. There are different possible explanations for the observed discrepancies regarding expression of Klotho in podocytes, such as the use of non-specific antibodies or contamination by tubular cells that can lead to the detection of a “false positive” signal.

Previous studies have demonstrated that Klotho can play a vital role also in tissues where its expression is close to undetectable, such as in osteocytes^29–31^. Therefore, we wanted to explore if the potential low-grade expression of Klotho in podocytes could still have a biological relevant role. Characterization of mice with a targeted deletion of Klotho in podocytes (Pod-KO) revealed no discernable abnormalities in glomerular structure or function. Furthermore, Pod-KO mice challenged with NTS-induced glomerulonephritis developed a similar degree of glomerular injury and proteinuria as WT mice, as evidenced by quantification of albuminuria and histological examination. Together, these data suggest that the protective effects of Klotho against glomerular injury observed in previous studies cannot be attributed to the local expression of Klotho in podocytes but is rather due to the effects of Klotho derived from other cell types. To address this hypothesis, we generated two novel transgenic mouse lines overexpressing full-length human Klotho in podocytes (Pod-hKL) or in hepatocytes (Alb-hKL), which do not express Klotho endogenously. The lack of protection afforded by overexpression of Klotho in podocytes when mice were challenged with NTS-induced glomerulonephritis confirms that locally expressed Klotho is unlikely to have a glomeruloprotective role. In contrast, mice overexpressing Klotho in hepatocytes (Alb-hKL) had significantly less glomerular injury after NTS challenge, supporting the notion that the beneficial role of Klotho in the context of glomerular injury is mediated via systemic effects from its soluble form.

Despite a rather extreme elevation in blood concentrations of Klotho in the Alb-hKL mice (up to 14,000-fold increase compared to WT mice), Fgf23 concentrations were only increased by 2-fold with no changes in serum or urine phosphate or calcium compared to WT mice. This is in stark contrast with results from previous studies of mice overexpressing soluble mouse Klotho by injection of adeno-associated virus, which resulted in up to a 450-fold increase in serum Fgf23 and marked hyperphosphaturia leading to hypophosphatemia^52^. Similarly, in a patient with a translocation causing increased Klotho expression serum FGF23 was markedly increased, and serum phosphate was low due to renal phosphate wasting^53^. The reason why there was no increase in Fgf23 or altered phosphate handling in our transgenic mice is unclear, but it could speculatively be due to lack of cross reactivity between human soluble Klotho and mouse Fgf23 and/or Fgfr1. Unfortunately, other studies overexpressing human Klotho did not report on serum Fgf23, phosphate, or calcium levels^54^. The close interdependence of FGF23 and Klotho often makes it difficult to discern FGF23-mediated effects from the effects mediated by soluble Klotho alone. In our study, the very mild increase in Fgf23 levels and the lack of hypophosphatemia suggests that the phenotypic differences in response to glomerular injury is indeed caused by soluble Klotho, and not by elevated Fgf23 and/or lower phosphate.

Of note, despite the extreme elevation in serum concentrations of Klotho there was no increase in urinary Klotho in Alb-hKL mice. Conversely, Pod-hKL mice had higher urinary Klotho concentrations, but unchanged serum concentrations. From these observations we conclude that (i) there is no substantial passage of Klotho over the glomerular filtration barrier, nor an active transcellular transport in the tubuli that is proportionate to the serum concentrations^8^, in healthy mice or in mice with glomerular injury and marked proteinuria, and (ii) the renoprotective effects of soluble Klotho appear to be mediated from the blood/systemic side, and not from the urinary side.

Using an unbiased approach, we found that the glomeruloprotective properties of soluble Klotho in the NTS model is, at least partly, mediated via modulation of ER stress/UPR. Using kidney biopsies from patients with diabetic nephropathy and precision-cut kidney slices from human kidney tissue we demonstrate that these observations translate well to a human context. The involvement of protein misfolding and maladaptive ER stress is well-established in the pathophysiology of diabetic nephropathy and other renal diseases^55,56^, and specific inhibition of CHOP was recently shown to reduce glomerular and tubulointerstitial damage in an animal model of diabetic nephropathy^57^. Moreover, our data suggest that soluble Klotho attenuates ER stress systemically, rather than specifically in the glomeruli. In support of this hypothesis, previous studies have suggested that Klotho modulates ER stress also in tubular epithelial cells and monocytes^58–60^. The exact mechanism by which soluble Klotho modulates ER stress and UPR, and the putative intermediate effector(s) remain unknown and is a topic for further studies. Importantly, pathways related to Wnt, VEGF, and insulin signaling were also modified by Klotho overexpression and are potentially part of the renoprotection (Supplementary Fig. 7). In contrast, Nrf2 and Trpc6 were not differentially expressed in Alb-hKL mice, suggesting that they are not involved in Klotho’s modulation of injury response, at least not in the NTS model.

In summary, we demonstrate that Klotho is not expressed in podocytes and that its specific deletion or overexpression in podocytes does not affect glomerular function, or susceptibility to injury. Moreover, we demonstrate that soluble Klotho derived from extraglomerular sources regulates the ER stress response which protects glomerular integrity and function. Future studies should determine the exact mechanism of action, and examine whether pharmacological treatment with soluble Klotho, or restoration of endogenous Klotho expression, could help maintain an appropriate response to ER stress, which has a critical role in the onset and progression of many types of kidney disease.

## Methods

### Study approval

The use of human material for this study was approved by the Ethical Review Board in Stockholm, Sweden, archive numbers 2010/579-31 and 2016/615-32. The study design and conduct for the human samples are compliant with the ethical permits and the Declaration of Helsinki. For mouse work, all experimental protocols were approved by The Ethical Committee for Research Animals, Linköping, Sweden (archive number DNR41-15 and DNR1336-19). All animals, unless specified otherwise, were on C57BL6/J background, housed in standard, single-ventilated cages with 12 h light–12 h dark cycle and had ad libitum access to water and chow. The house temperature was maintained as 20 ± 2 °C and the relative humidity was kept as 50 ± 5%.

### Transgenic mouse lines

Mice with a podocyte-specific Klotho deletion were generated using Cre-LoxP recombination as described previously^25^ (supplemental Fig. 1). The genotyping was done by PCR using genomic DNA extracted from ear biopsies. Primers for genotyping were: *Klotho*-LoxP-F: 5′ttgtcaatatgtaaataatttgagcagtaggg3′, *Klotho*-LoxP-R: 5′gttgttgaaagagggagctagtggtagtta3′. The Gt(ROSA)26Sor^tm1(CAG-KLOTHO-T2A-EGFP)^/J mice were crossed with a albumin-cre or podocin-cre line to activate Klotho expression specifically in hepatocytes or podocytes, respectively. Breeding and genotyping were done according to standard procedures.

### Anti-GBM glomerulonephritis model

Animals between 8 and 13 weeks old were pre-immunized subcutaneously with 400 µl/kg of sheep IgG Freund’s complete adjuvant (F5881, Sigma-Aldrich). Four days later, glomerulonephritis was induced by intravenous injection of 130 μl of nephrotoxic serum (NTS) purchased from Probetex (PTX-001S) and different batches were used for Pod-KO and Pod-/Alb-hKL mice. Experimental mice were maintained under standard animal house conditions in a conventional animal facility for 7 days. After that period, the animals were placed under isoflurane USP (Baxter) anesthesia to proceed to the organ collection. Histopathology of animals was evaluated by scoring at least 30 glomeruli/mice as “normal” or “abnormal” (including segmental sclerosis, crescents, and necrosis). To assess the extent of tubular cast formation, the whole cross-section was evaluated. The score was determined as follows: 0, no casts; 1, >0–20%; 2, 20–40%, 3, 40–60%; 4, 60–80%; 5, >80% of the tubules are affected.

### Cell culture

Mouse podocytes were cultured as previously described^61^. Briefly, podocytes were grown under permissive conditions at 33 °C in RPMI 1640 media containing 10% FBS, 10 U/ml interferon-γ. The cells were treated with 5 μg/ml Tunicamycin (#3516, Tocris Bioscience) and/or 0.05 μg/ml recombinant human Klotho (#5334-KL, R&D Systems) for 16 h prior to lysis using RIPA lysis buffer (ThermoFisher Scientific) containing protease and phosphatase inhibitors (Roche).

### In situ hybridization

In situ hybridization was carried out on paraffin-embedded sections using RNAscope™ (Advanced Cell Diagnostics) according to manufacturer’s instruction. Kidneys from 8-week-old 129S2/SvPasCrl, CD1(ICR), and C57Bl6/J males were stained using probes against the sequence 879-1844 and 49-1754 of the mouse Klotho and Nephrin genes, respectively. For sections from human kidney probes against the sequence 797-1768 of human Klotho gene was used.

### Quantitative PCR

Glomeruli were isolated from human and mouse kidneys as published previously^62,63^. Mouse podocytes from tdTomato mice were isolated as described previously^33^. Total RNA was extracted using RNeasy mini kit (Qiagen). First-strand cDNA synthesis was carried out using the iScript cDNA Synthesis Kit (Bio-Rad). For real-time qPCR analysis, theCFX96Real-Time PCR Detection System and iQ SYBR Green Supermix (Bio-Rad) were used. The relative gene expression was calculated with the 2^−ΔΔCq^ method normalizing the gene of interest to housekeeping gene in the same sample. Data are presented as relative fold-change. The used primers were hu*28S*-F: 5′ttgaaaatccgggggagag3′, hu*28S*-R: 5′acattgttccaacatgccag3′, hu*KL*-F: 5′tccatctgggatacgttcaccc3′, hu*KL*-R: 5′tgtcgcggaagacgttgttg3′, hu*NPHS1*-F: 5′gagagccccattcaaaggct3′, hu*NPHS1*-R: 5′agaaggagctcacggtttcg3′, hu*HSPA5*-F: 5′cgggcaaagatgtcaggaaag3′, hu*HSPA5*-R: 5′ttctggacgggcttcatagtagac3′, hu*HSP90B1*-F: 5′ggatggtctggcaacatgga3′, hu*HSP90B1*-R: 5′ccgaagcgttgctgtttcaa3′, hu*uXBP1*-F: 5′tccgcagcactcagactacg3′, hu*u/sXBP1*-R: 5′agttgtccagaatgcccaaca3′, hu*sXBP1*-F: 5′ctgagtccgcagcaggtg3′, hu*DDIT3*-F: 5′ggagcatcagtcccccactt3′, hu*DDIT3*-R: 5′tgtgggattgagggtcacatc3′, mm*Gapdh*-F: 5′tgttcctacccccaatgtgt3′, mm*Gapdh*-R: 5′tgtgagggagatgctcagtg3′, mm*Nphs1*-F: 5′cttttggcttcgctgtcacc3′, mm*Nphs1*-R: 5′aaggcccagattgcatcgta3′, mm*Kl*-F: 5′tctcaagaattcataatggaaacc3′, mm*Kl*-R: 5′cagaaagtcaacgtagaagagtcct3′.

### Immunohistochemistry and Immunofluorescence

Kidneys were fixed with 4% paraformaldehyde and subsequently embedded with paraffin. Paraffin-embedded kidney sections (4μm) were processed for the following staining. For immunohistochemical staining of GRP78/BiP and CHOP, a HIER was performed using a Decloaking Chamber (Biocare Medical) set for 5 min at 110 °C in Citrate buffer pH 6 (C-999, Sigma-Aldrich) followed by a 15 min blocking step using Biocare Medical Background Sniper. The primary antibodies: mouse monoclonal anti-CHOP (clone L63F7; #2895, 1:400, Cell Signaling) and rabbit monoclonal anti-GRP78/BiP (clone [EPR4041(2)]; ab108615, 1:2000, Abcam) were diluted in Renior Red diluent (PD9004M, Biocare Medical) and incubated at 4 °C overnight in a humid chamber. For detection, the Mach-1 Universal HRP-Polymer Kit was used (MIU539L10, Biocare Medical) according to protocol. For all the slides, nuclear counterstaining was performed using Mayer hematoxylin solution. Tissue sections were observed on a Zeiss Observer Z1. For immunofluorescent studies, after HIER treatment with Tris-EDTA pH9 (Citrate pH 6 for anti-GFP staining) for 20 min at 100 °C, sections were blocked using 10% goat serum + 0.1% Tween 20 solution for 1 h at room temperature. sections were incubated with anti-nephrin (BP5030; 1:200, Origene), anti-podocin (P0372; 1:500, Sigma-Aldrich) anti-vimentin (V5255; 1:400, Sigma-Aldrich), anti-WT1 (ab89901; 1:100, Abcam), anti-Klotho (clone Km2076; 1:200, Trans Genic Inc.), anti-αSMA – Cy3™ (C6198; 1:1000, Sigma-Aldrich), anti-GFP (ab290; 1:2000, Abcam) at 4 °C overnight, anti-synaptopodin (#61094; 1:700, PROGEN Biotechnik GmbH) for 1 h at room temperature and fluorescein-labeled Lotus Tetragonolobus Lectin (FL-1321; Vector Labs) for 1 h at 37 °C. Alexa Fluor conjugated secondary antibodies were used for visualization (Invitrogen). All the slides were co-stained with Hoechst 33342 (H3570, 1:10,000, Life Technologies) for 10 min at room temperature. Sudan black B (0.1% in 70% Ethanol, #199664, Sigma-Aldrich) solution was applied for 25 min at room temperature before mounting in Dako fluorescent medium (S3023, Agilent).

Immunofluorescent studies from Supplementary Fig. 2. were performed using frozen mouse kidney tissue preserved in Tissue-Tek™ O.C.T compound (#4583, Sakura, CA, USA). Samples were fixed in ice-cold acetone for 20 min and blocked with 5% normal donkey serum (C06SB, Bio-Rad) for 1 h at room temperature. Sections were incubated with anti-Klotho (AF1819; 1:200, R&D Systems) overnight at 4 °C and anti-synaptopodin (#61094; 1:700, PROGEN Biotechnik GmbH) for 1 h at 37 °C. Alexa Fluor conjugated secondary antibodies were used for visualization (Invitrogen, Carlsbad, CA, USA). All the slides were co-stained with Hoechst 33342 (H3570, 1:10,000, Life Technologies) for 10 min at room temperature.

Immunofluorescent studies from Supplementary Fig. 3. were performed following the method described in Kim et al. Briefly, frozen sections were fixed in prechilled 100% acetone for 10 min at −20 °C and blocked with 3% BSA and 3% normal donkey serum in PBS at room temperature for 1 h. Slides were stained with goat anti-synaptopodin (sc21537, 1:20, Santa Cruz Biotechnology) at 37 °C for 1 h, followed by rat monoclonal anti-klotho antibody (Km2076, 1:20, Trans Genic Inc.) at 37 °C for 1 h and at 4 °C overnight. Alexa Fluor conjugated secondary antibodies were used for visualization (Invitrogen, Carlsbad, CA, USA). All the slides were co-stained with Hoechst 33342 (H3570, 1:10,000, Life Technologies) for 10 min at room temperature.

### Optical Clearing and Imaging

A slightly modified version of a previously published protocol was used^64^. In brief, formaldehyde/formalin fixed kidneys were embedded in 3% agarose in DI water and then sectioned to 200 µm thickness using a Vibratome. Slices were then incubated in clearing solution (200 mM boric acid, 4% SDS, pH 8.5) at 50 °C overnight. Sections were washed in PBST (0.1% Triton-X in 1X PBS, pH 7.4) for 10 min before incubation in a sheep polyclonal antibody against nephrin (AF4269, R&D systems) diluted at 1:100 and a rat monoclonal against Klotho (KM2076, Trans Genic Inc.), diluted at 1:200 in 1X PBS with 0.3% Triton X-100 (PBST) at room temperature overnight. After primary antibody incubation, samples were washed in PBST for 5 min at room temperature and were then incubated in a donkey anti-sheep secondary antibody conjugated to Alexa-488 (#713-005-147, Jackson Immuno) diluted at 1:100 and a donkey anti-rat secondary antibody conjugated to Alexa-647 (#712-605-153, Jackson Immuno) diluted at 1:100 in PBST at room temperature overnight. Samples were incubated in 80% wt/wt fructose (1 mL of dH20 added to 4 g of fructose with 0.25% 1-Thioglycerol) at 37 °C for 15 min and then mounted in a MatTek dish with a cover slip on top prior to imaging using a Leica SP8 3X gSTED system using either a 20 × 0.75 NA oil immersion objective or a 100 × 1.4 NA oil immersion objective.

### Transmission electron microscopy

TEM was carried out by the electron microscopy unit core facility (EMil) at Karolinska Institutet, Huddinge University hospital per their standard protocols.

### Western blot analysis

For Western blot analysis, glomeruli and rest of kidney fractions were lysed in RIPA lysis buffer (ThermoFisher Scientific) containing protease and phosphatase inhibitors (Roche) for 15 min on ice. The protein content of the samples was determined using Pierce™ bicinchoninic acid assay kit (ThermoFisher Scientific). Equal amounts of protein were separated on sodium dodecylsulfate–polyacrylamide gels, blotted onto polyvinylidene fluoride membranes and blocked in 5% nonfat dry milk in triethanolamine-buffered saline with 0.1% Tween-20. Western blots were probed with antibodies directed against klotho (Rat monoclonal Km2076; 1:100 ON at 4 °C, Trans Genic Inc.), podocin (Rabbit polyclonal P0372; 1:1000 for 1 h at RT, Sigma-Aldrich), calnexin (rabbit polyclonal ab10286; 1:1500 for 1 h at RT, Abcam), GRP78/BiP (Rabbit polyclonal ADI-SPA-826; 1:1000 ON at 4 °C, EnzoLife Sciences) and LC3B (Rabbit polyclonal NB100-2220; 1:1000 ON at 4 °C, Novus Biologicals). Secondary antibodies were purchased from ThermoScientific. For detection method, we used Pierce^TM^ ECL western blotting substrate (#32209, ThermoFisher) and the Licor Odyssey Fc imaging system (Licor).

### Biochemistry

Serum and urine calcium, phosphate, and creatinine were measured using colorimetric kits (#K380, K410, K625, respectively, Biovision). Intact FGF23 was measured using an intact FGF23 ELISA kit (#60-6800, Triolab). The urinary albumin/creatinine ratio was measured by using the commercial kits Albuwell (#1011, Exocell) and Quantichrome creatinine assay kit (#DICT-500, Bio Assay Systems). The fractional excretion of phosphorus (FEPi) and calcium (FECa) were calculated as [(urine mineral*serum creatinine)/(serum mineral*urine creatinine)].

### mRNA isolation and sequencing

Glomeruli were isolated from mouse kidneys as published previously^62,63^. Total RNA was extracted using TRIzol^TM^ reagent (ThermoFisher Scientific). RNA quality was assessed by determining its RNA integrity number (RIN). mRNA-seq libraries were constructed using a TruSeq RNA SamplePrep kit (Illumina) according to the manufacturer’s instructions. Sequencing was conducted for 75 cycles on Illumina Nextseq 550 sequencer according to the manufacturer’s instructions.

### Processing of mRNA-seq reads and downstream analysis

Bcl files were converted and demultiplexed to fastq using the bcl2fastq v2.20.0.422 program. STAR 2.7.5b^65^ was used to index the mouse reference genome (mm10/GRCm38) and align the resulting fastq files. Mapped reads were then counted in annotated exons using featureCounts v1.5.1^66^. The annotations and reference genome were obtained from Ensembl. The count table from featureCounts was imported into R/Bioconductor and differential gene expression was performed using the EdgeR^67^ package and its general linear models pipeline. Both conditions were analyzed by using four biological replicates. For the gene expression analysis, genes that had 1 count per million in 3 or more samples were used and normalized using TMM normalization. Gene expression analysis was also performed using voom/limma. The gene counts were transformed to log2-counts per million using voom^68^ with samples weights and subsequent differential gene expression analysis was performed in limma^69^. Genes with an FDR adjusted *p* value <0.05 were termed significantly regulated.

### Gene functional enrichment analysis and annotation

Gene functional enrichments were determined using the DAVID Bioinformatics Resources (v. 6.8)^70^. Annotation clusters identified by DAVID (clusters of related annotation terms) having an enrichment score of ≥1.3 were considered significant and a representative naming for the cluster was derived from the contained Gene Ontology terms and KEGG pathway analysis.

### Precision-cut kidney slices

A modified version of a previously published protocol was used^49^. Human kidney pieces were decapsulated and embedded in 3% agarose prewarmed at 40 °C. Slices were cut in ice-cold DPBS using a vibrating microtome Compresstome® VF310-0Z (Precisionary Instruments). Immediately after, the 300-μm-thick slices were pre-incubated containing Williams’ Medium E + GlutaMAX™-I (Gibco™, ThermoFisher Scientific) supplemented with 25 mM D-glucose and 1% Penn-Strep (10,000 U/mL, Gibco™, ThermoFisher Scientific) for 1 h at 37 °C under 80% O_2_ and 5% CO_2_ atmosphere to allow the slices to restore their ATP levels^71^. Slices were then cultured for an additional 24 h with 1 μM tunicamycin^72^ (#3516, Tocris Bioscience) and 0.05 μg/mL recombinant human Klotho (#5334-KL, R&D Systems) at 37 °C, 80% O_2_ and 5% CO_2_ on an orbital shaker at 80 RPM. Slices were harvested in 10% formalin solution (HT501128, Sigma-Aldrich) for histology analysis and in RNAlater for RNA isolation. All conditions were carried out as three technical replicates.

### Statistics and reproducibility

Statistical analysis was performed with GraphPad Prism software (La Jolla, CA). For in vivo, in vitro, and ex vivo experiments, data were analyzed using Student’s *t*-test or one-way ANOVA followed by Tukey’s multiple comparisons test when appropriate. Urinary albumin-creatinine ratio time course was analyzed using a mixed model ANOVA. Pearson correlation was used to analyze the association between the level of klotho transcript expression and ER/UPR transcripts expression in human. A *p* value inferior to 0.05 was considered statistically significant. All data are presented as mean ± SD.

### Reporting summary

Further information on research design is available in the Nature Portfolio Reporting Summary linked to this article.

## Supplementary information

Supplementary information

Description of Additional Supplementary Data

Supplementary Data 1

Reporting summary

## Data Availability

The data supporting the findings of this study are openly available in repository Gene Expression Omnibus (accession number GSE195641). The supplementary information file contains uncut gel images (Supplementary Figs. 8–13). The source data for all the graphs and charts are provided in an Excel file format in Supplementary Data 1.
