# Supplementary material for: Soluble Klotho protects against glomerular injury through regulation of ER stress response
Source: Commun Biol. 2023 Feb 22;6:208. doi: 10.1038/s42003-023-04563-1 (PMC9947099; doi:10.1038/s42003-023-04563-1)

Supplementary Figures

Supplementary Fig. 1. Single-cell RNA sequencing data showing cluster specificity extracted from He *et al.* 2021. DTL: descending thin limb of Henle's loop; GEC: glomerular endothelial cells; MLC: Mesangial-like cells; PTC: Proximal tubular cells; T+NK: T + natural killer lymphocytes; cTAL + CD : collecting ducts; DCT: distal convoluted tubules; EC: endothelial cells; PEC: glomerular parietal epithelial cells; cTAL: cortical thick ascending limb of Henle's loop. Database available at https://patrakkalab.se/kidney/

Podocyte marker – Nephrin NPHS1

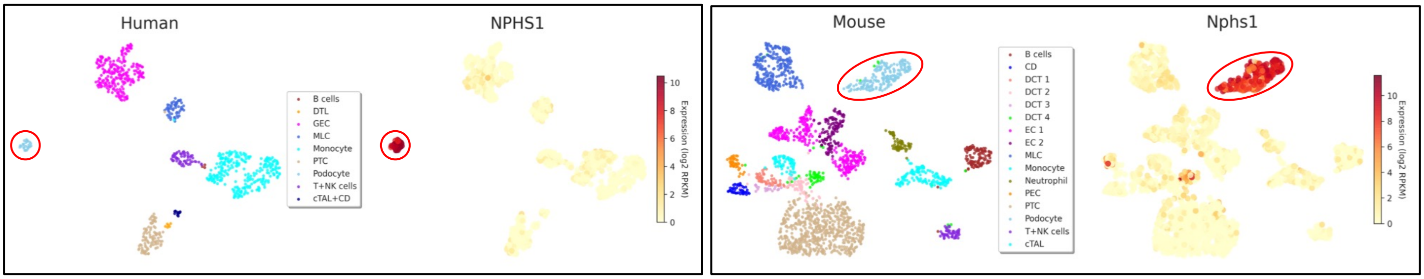

Distal Tubule marker – E-Cadherin CDH1

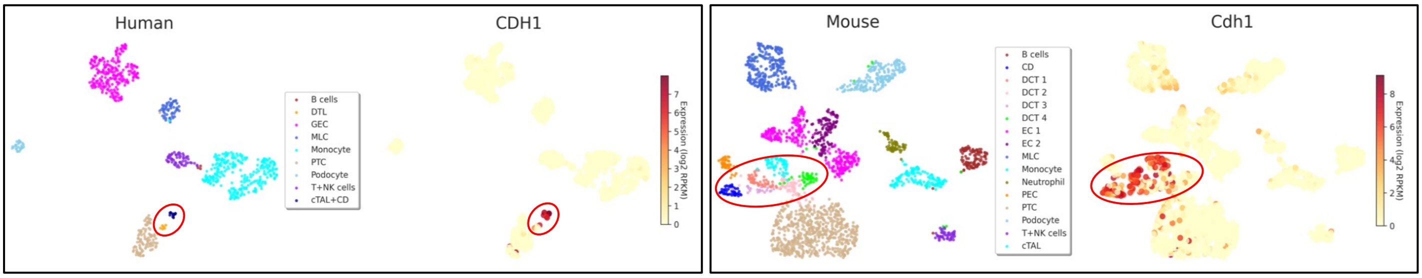

Proximal Tubule marker – Sodium-dependent phosphate transport protein 2A SLC34A1

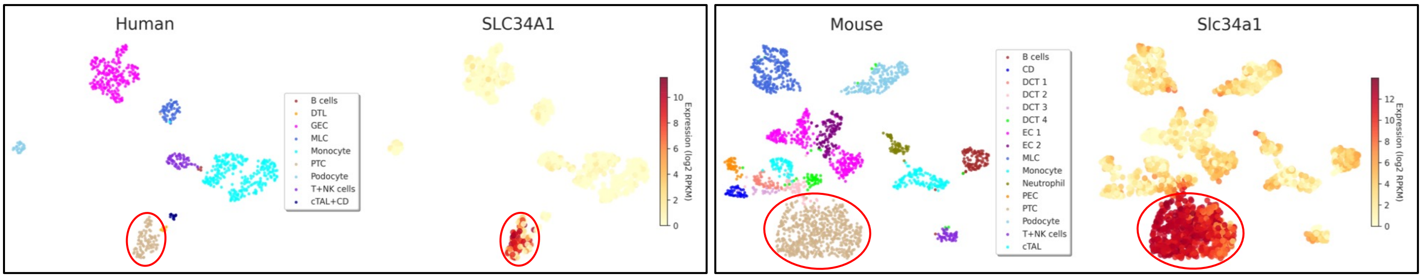

Supplementary Fig. 2. Immunofluorescence staining in paraffin-embedded human and mouse kidney using a different Klotho antibody (AF1819). Klotho staining (red) showed no colocalization with the podocyte marker Synaptopodin (green) in both human or mouse kidney. The specificity of Klotho AF1819 antibody was confirmed in the lower panel using β-actin-KO with complete deletion of Klotho. NHK: Normal human kidney, WT: Wild-type mouse, scale bars : 50 and 20 μm respectively.

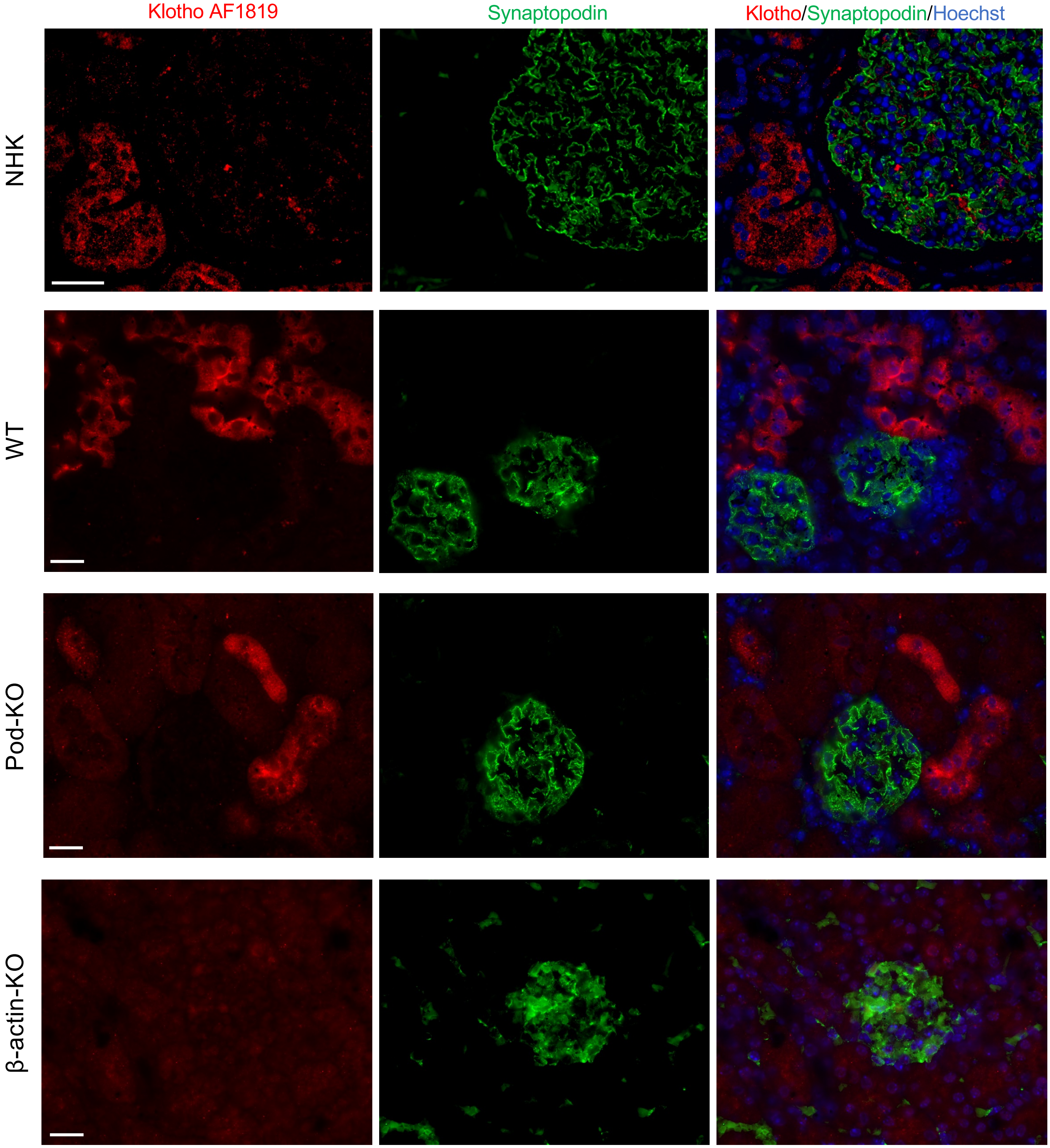

Supplementary Fig. 3. Immunofluorescence staining in frozen human and mouse kidney using the protocol described in Kim *et al.* 2017 using Klotho KM2076 antibody. Klotho (red) is co-labeled with the podocyte marker Synaptopodin (green) and no colocalization was detectable. NHK: Normal human kidney, WT: Wild-type mouse, scale bars : 50 and 20 µm respectively.

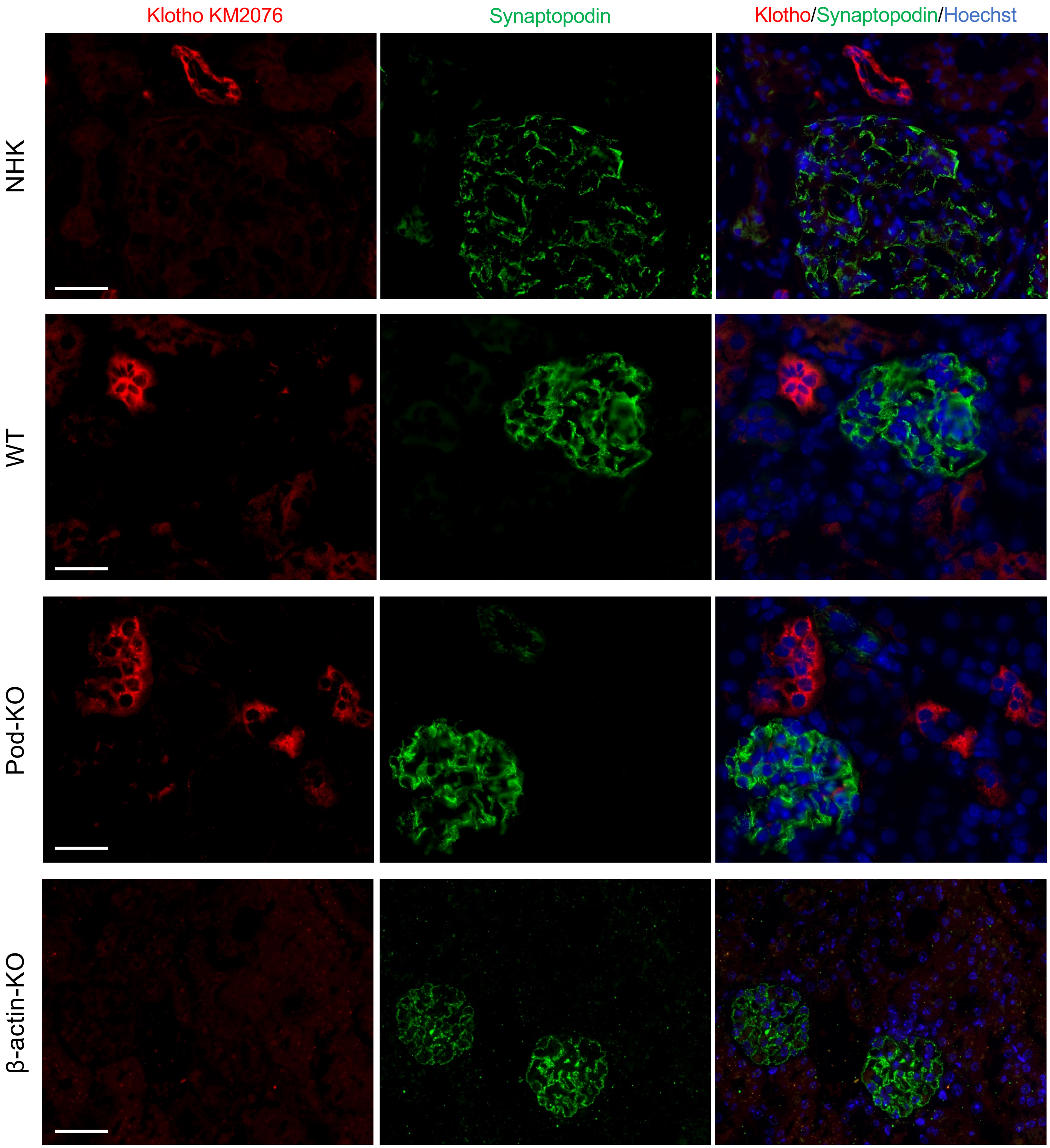

Supplementary Fig. 4. Generation of podocyte-specific Klotho knockout mouse line. (**a**) Schematic representation of the targeting strategy to generate conditional Klotho knockout mice. The top panel represents the wild-type allele, the targeting vector is depicted in the middle and the bottom panel shows the floxed allele with deleted Neo cassette. LoxP sites were inserted to surround exon 2 of Klotho gene and therefore enabled targeted deletion by Cre recombination (previously published1). (**b**) Representative PCR products from WT (370bp), Klotho$^{flox/+}$ (370 and 470bp), Klotho$^{flox/flox}$ (470bp) mice. (**c**) In situ hybridization staining for Klotho showing the specificity of the loxP-Cre recombination. The left panel revealed strong Klotho mRNA expression in distal tubuli, a weaker but substantial expression in proximal tubuli and no expression in glomeruli in WT mouse. In the middle panel, Klotho mRNA expression in proximal tubuli is no longer detectable as Klotho was specifically deleted in proximal tubuli using a Pepck-Cre deleter. The right panel demonstrated a complete loss of signal in β-actin-KO mice that are characterized by a global deletion of Klotho. Scale bar: 100 µm. (**d**) Schematic diagram of generating a conditional knockout mouse by breeding a podocyte-specific Cre mouse (top left) with a mouse carrying floxed Klotho gene (top right). Conditional knockout mice lack exon 2 of klotho gene. (**e**) The efficiency of cre-expression in podocytes was confirmed by staining of Gt(ROSA)26Sortm14(CAG-tdTomato)Hze/J mouse. Scale bar: 20 µm (**f**) No evidence of albuminuria was detected in 5-month old Pod-KO mice using SDS-PAGE gel. BSA (5µg) was used as a positive control.

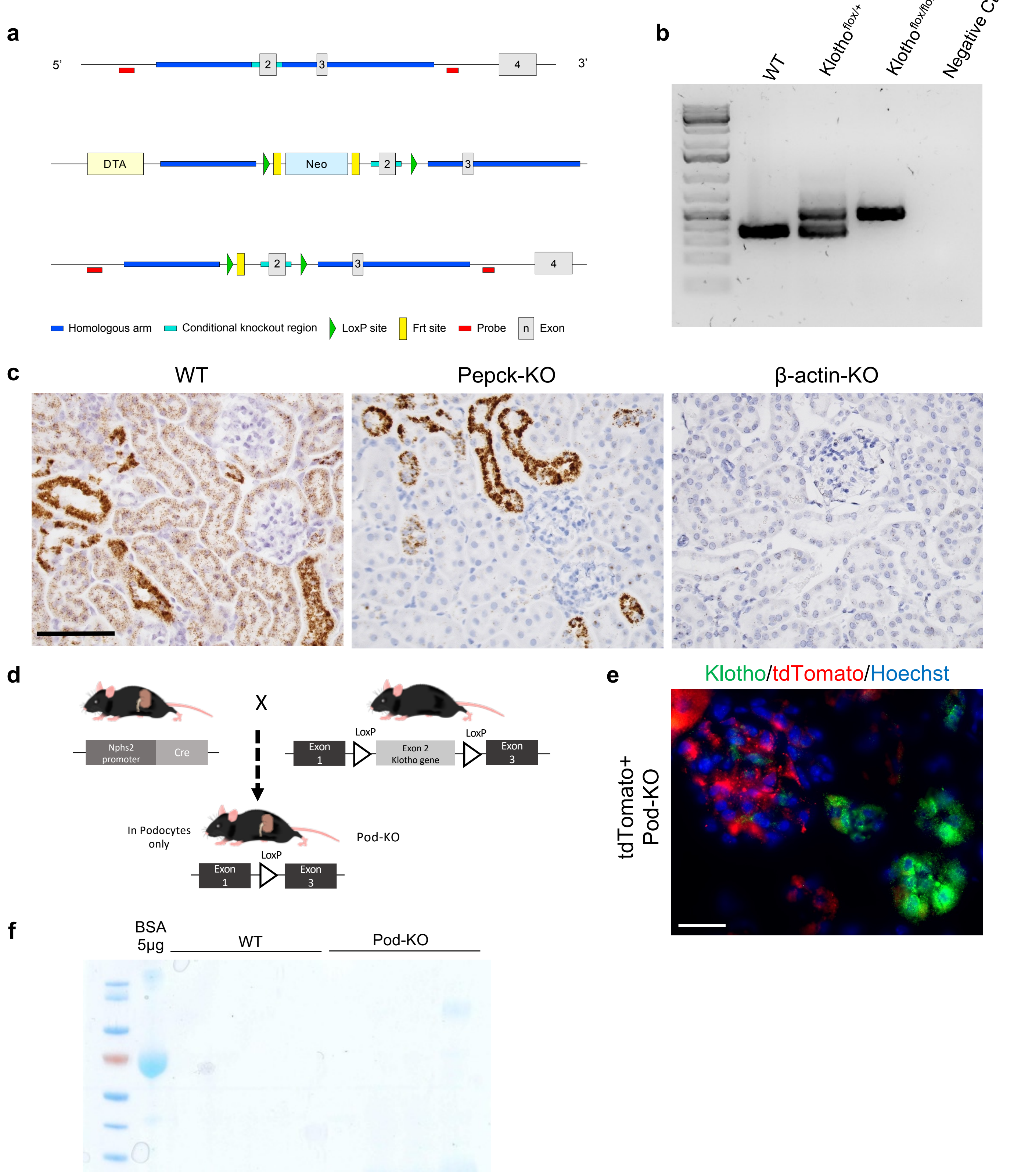

Supplementary Fig. 5. Immunofluorescent stainings for podocyte-specific markers nephrin, synaptopodin, vimentin and WT-1 show similar staining pattern in Pod-KO compared to WT mice. Scale bar: 20 µm

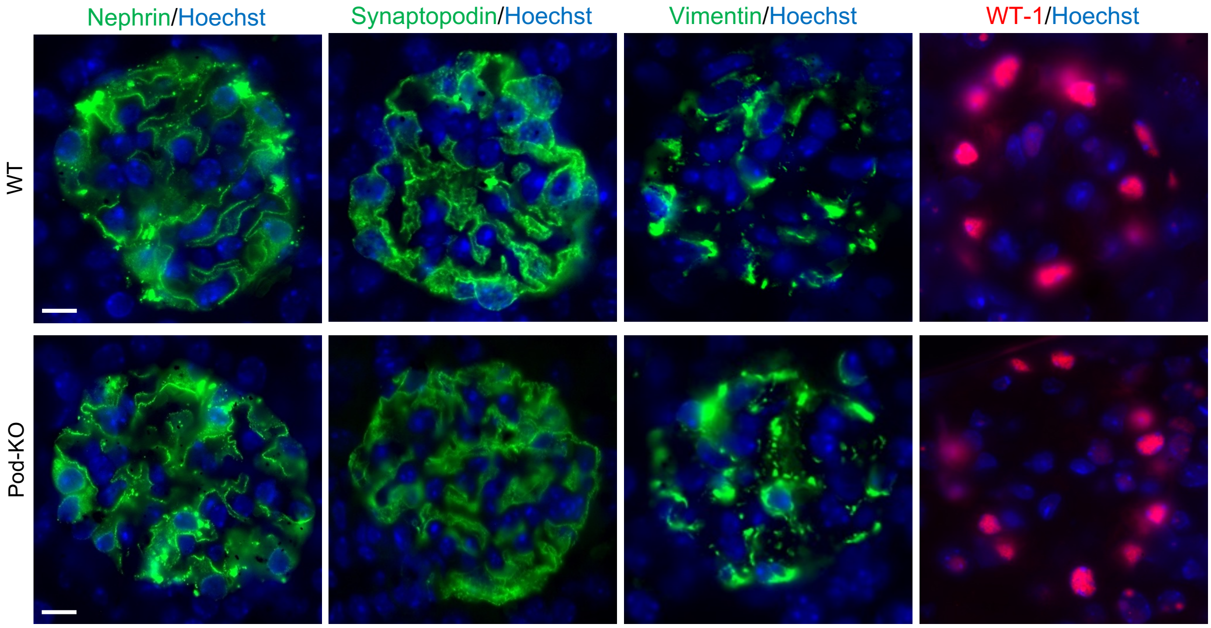

Supplementary Fig. 6. Klotho levels measured by Western Blotting in different strains (a) and in Alb-hKL compared to WT at baseline (b) and (c) transcript level of β-Klotho (*Klb*) in the liver at baseline (control) and after injury (NTS); *Gapdh* was used as housekeeping gene. No significant differences were measured.

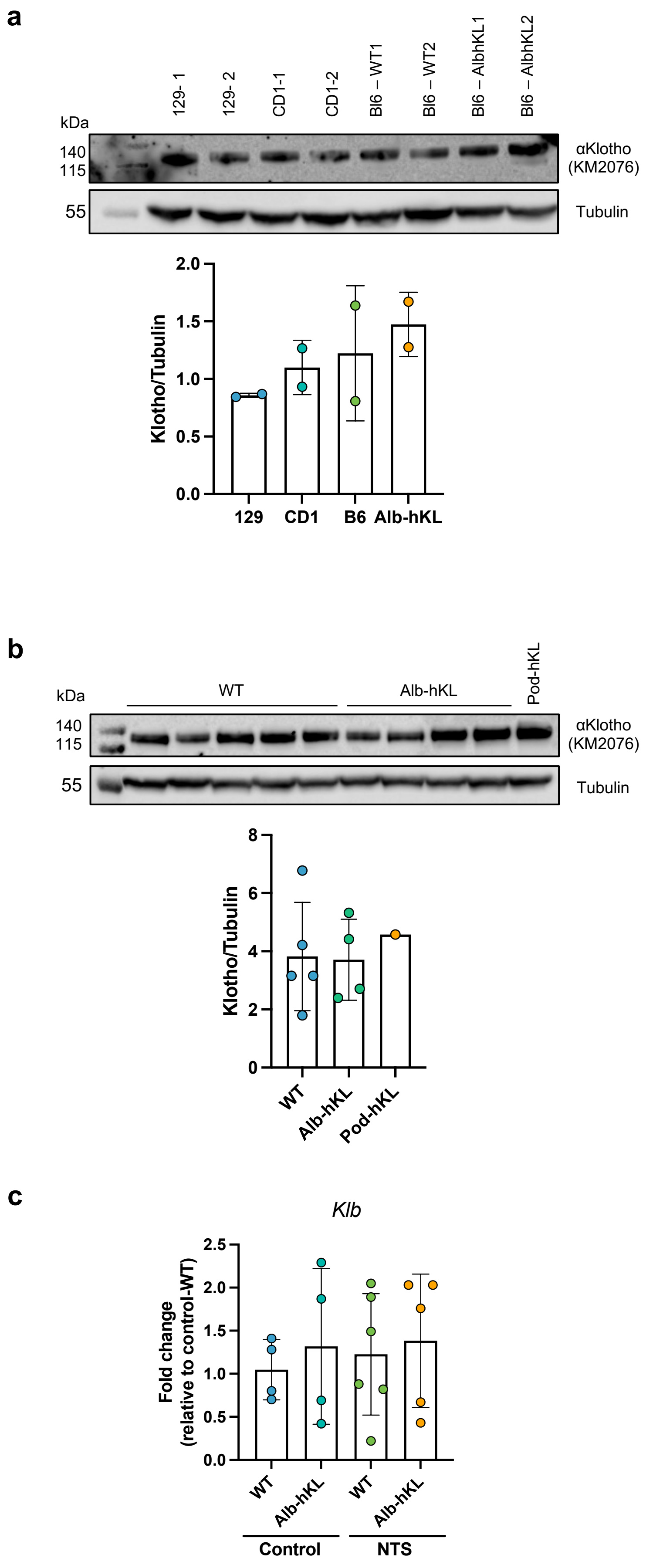

Supplementary Fig. 7. KEGG pathway analysis conducted on the down-regulated genes in the glomerular fraction from NTS-Alb-hKL vs. NTS-WT shown in Fig. 5. Only significant enrichments are shown. Red arrows highlight the KEGG pathways of interest.

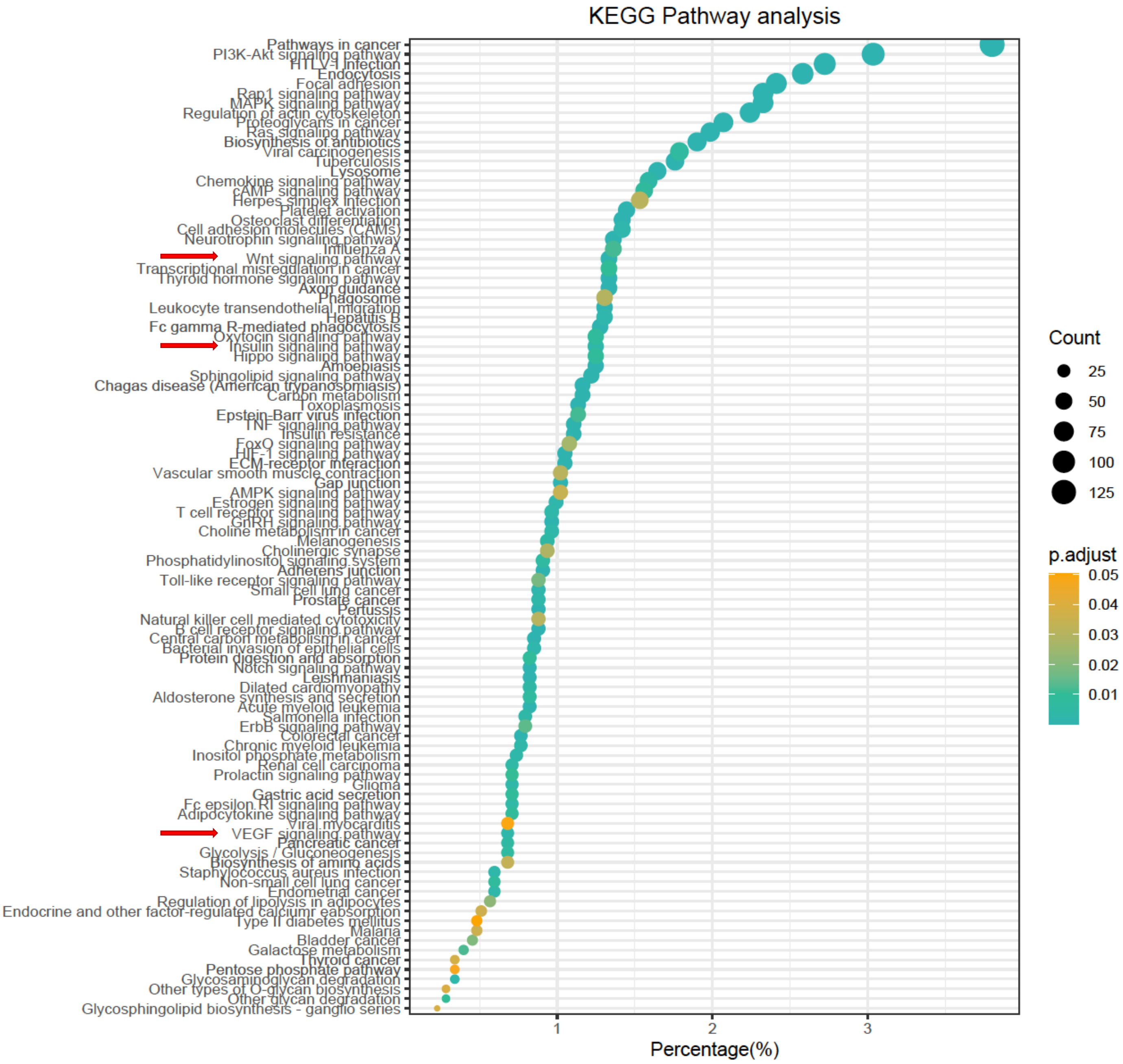

Supplementary Fig. 8. Uncut gel of Figure 1i-j.

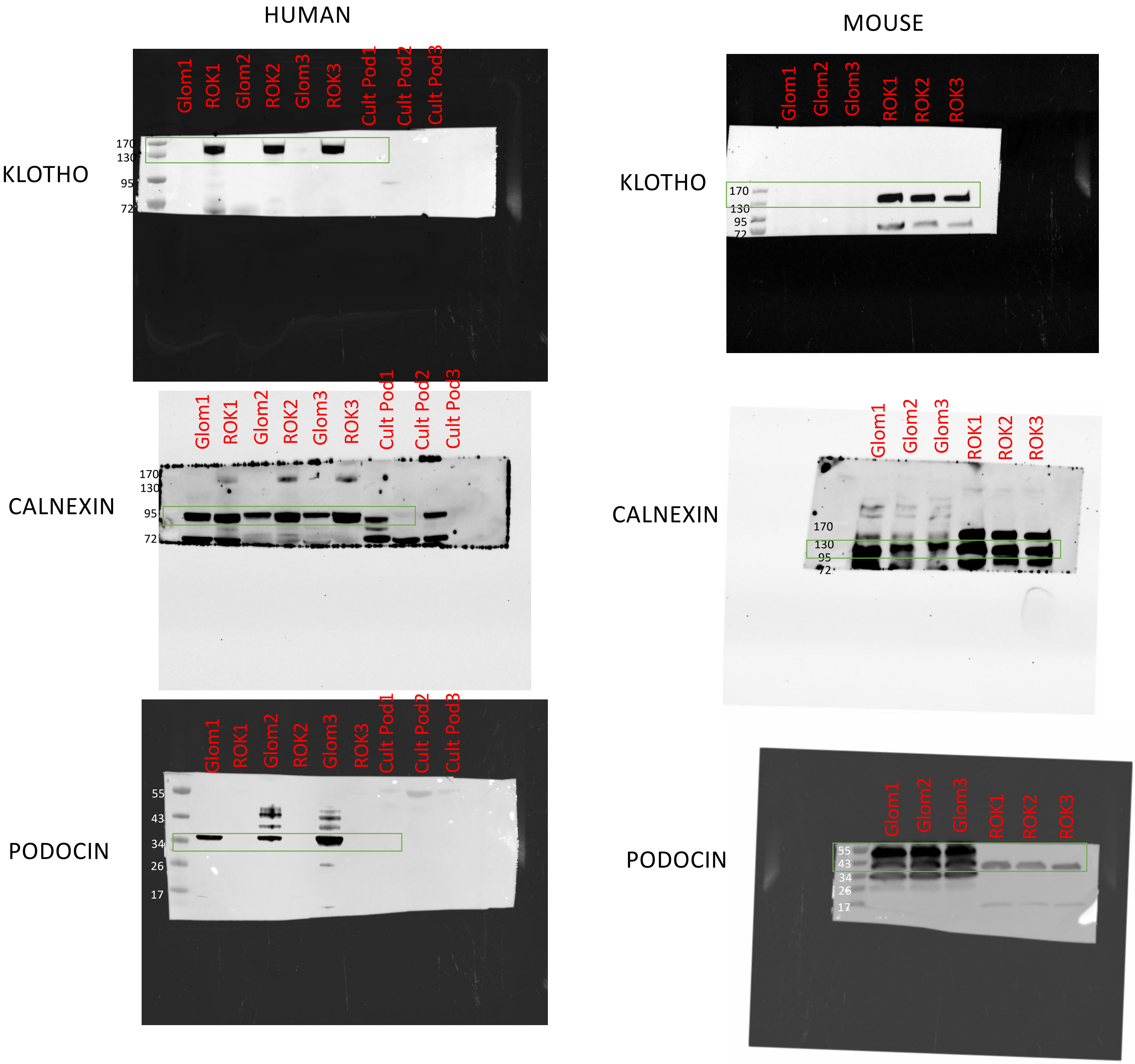

Supplementary Fig. 9. Uncut gel of Figure 3b.

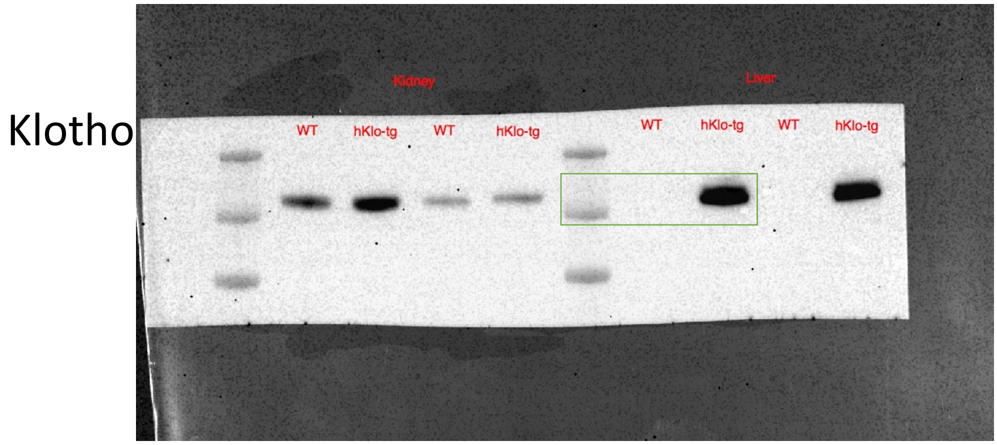

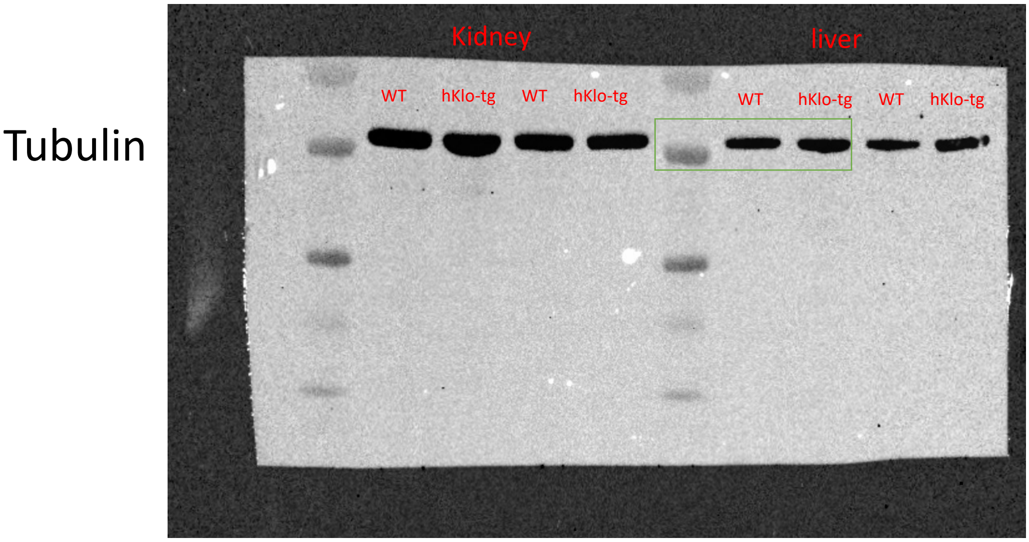

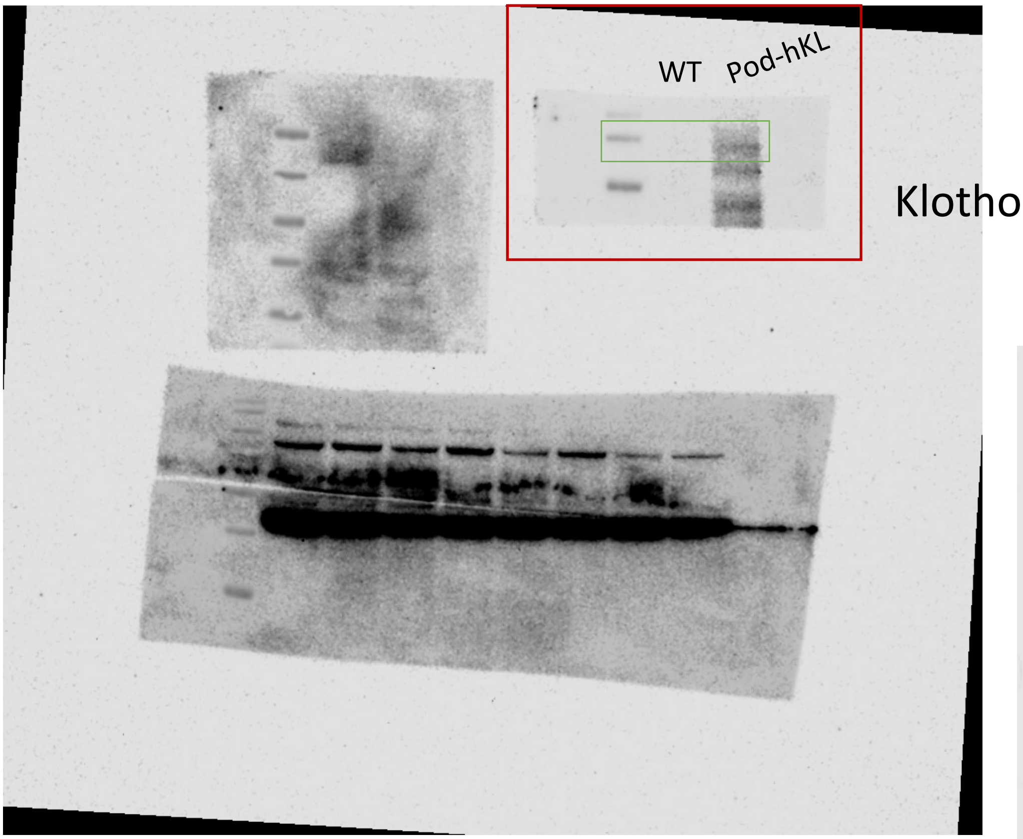

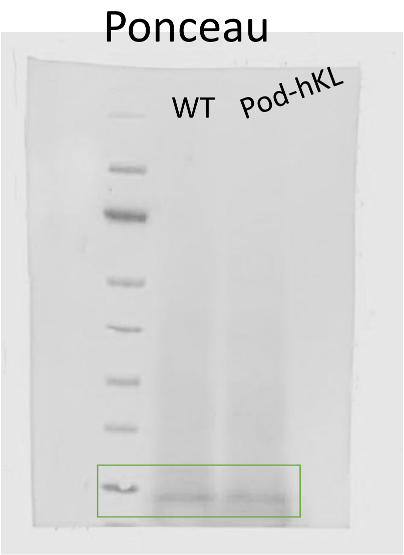

Supplementary Fig. 10. Uncut gels of Figure 5c-d used as representative pictures.

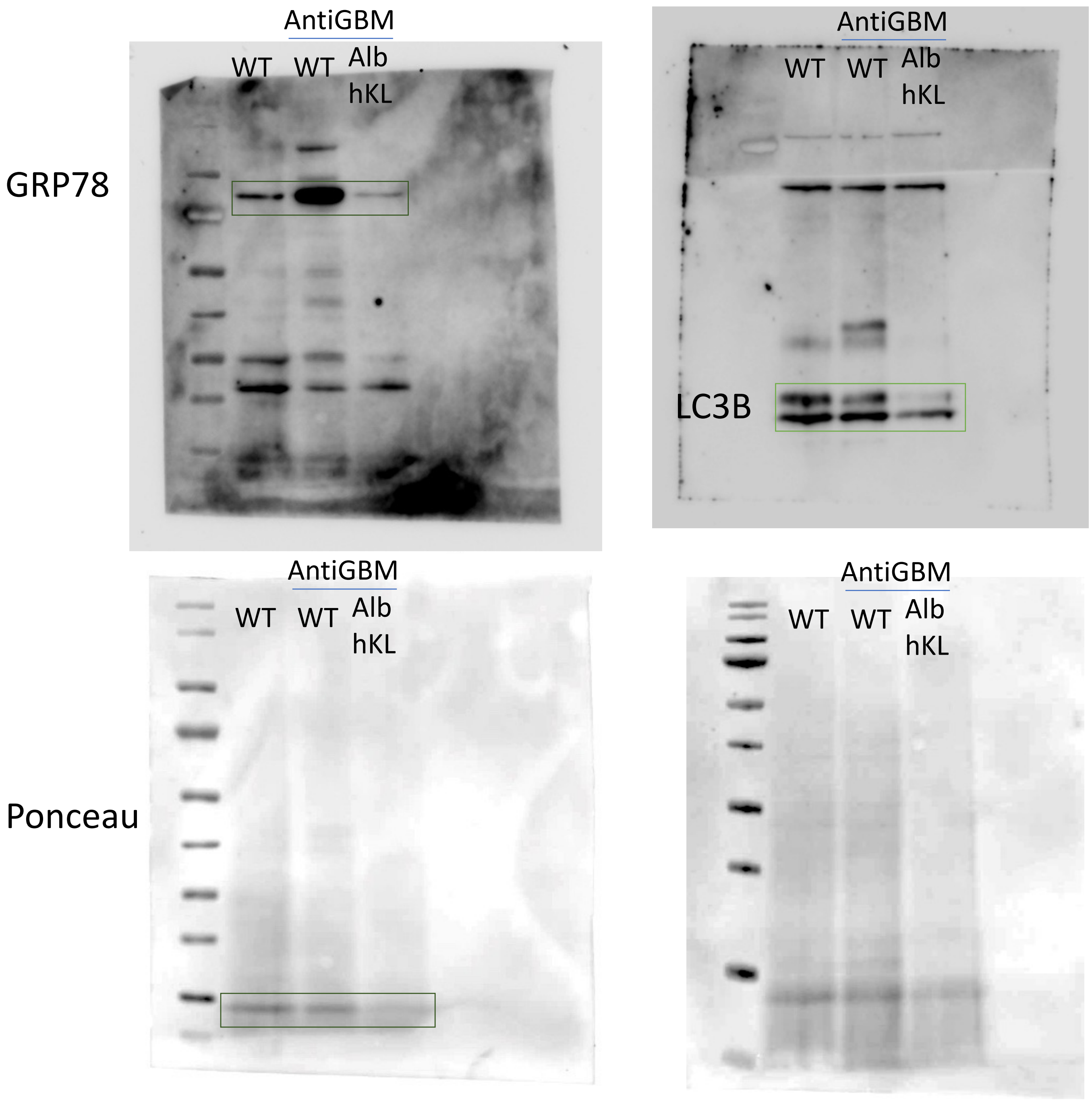

Supplementary Fig. 11. Uncut gels of Figure 5e-f used as representative pictures.

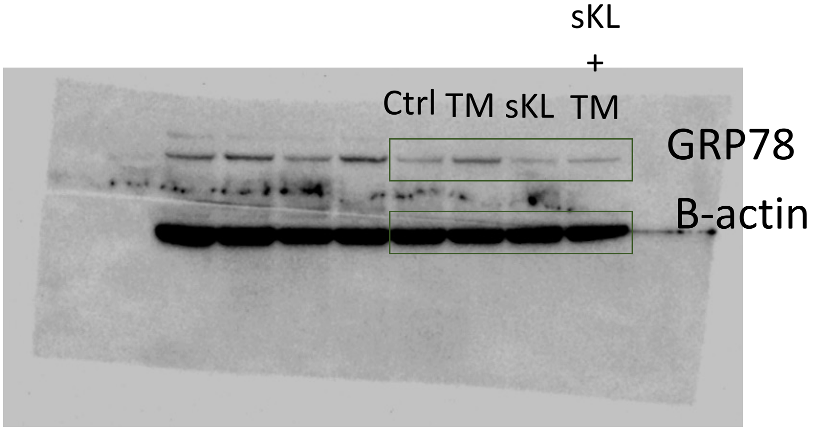

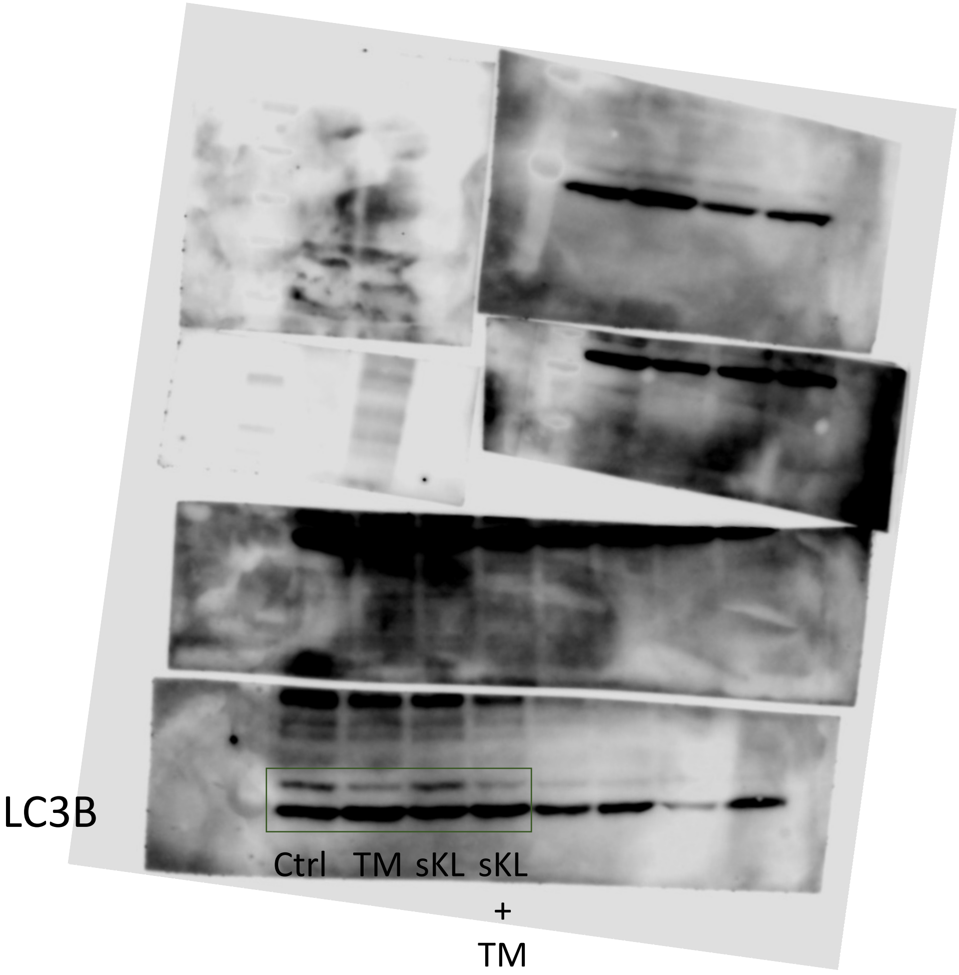

Supplementary Fig. 12. Uncut gels of Figure 5c-d used for quantification.

Glomerular fraction from mouse kidney for quantification Fig5C-D

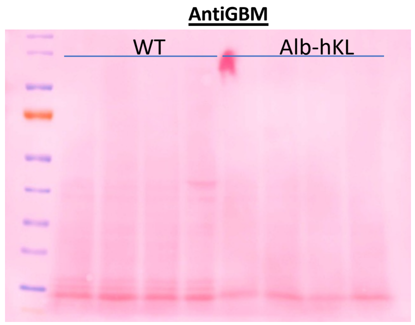

2021-04-12 4-12%Gel AntiGBM hKL

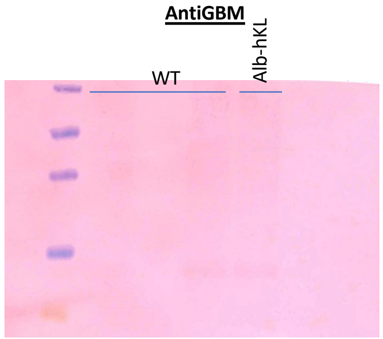

2021-03-26 4-12%Gel AntiGBM hKL

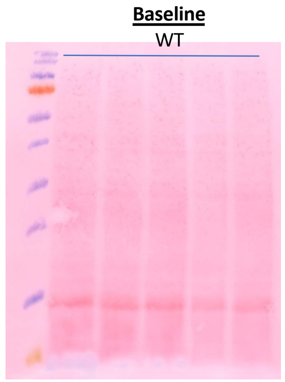

2021-04-13 4-12%Gel BL hKL

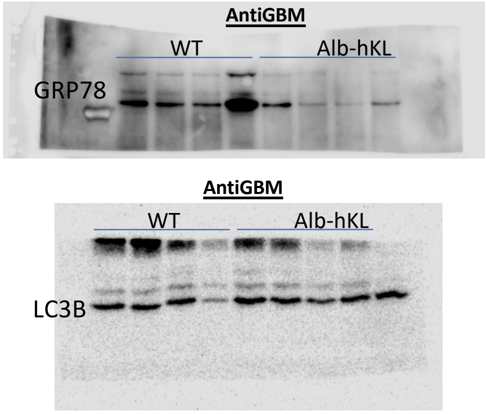

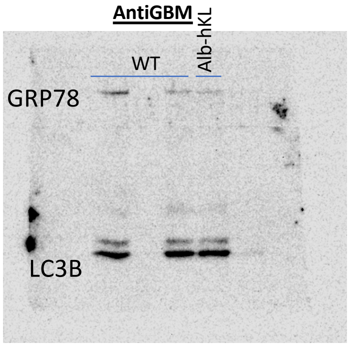

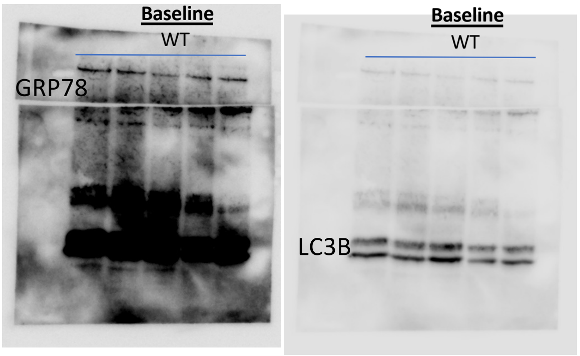

Supplementary Fig. 13. Uncut gels of Figure 5e-f used for quantification.

GRP78

Ctrl 1 TM 1 sKL 1 sKL+ TM 1

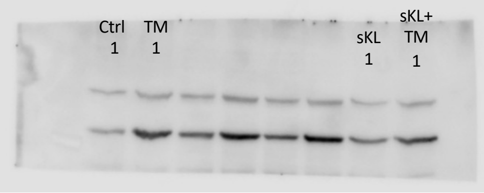

B-actin

Ctrl 1 TM 1 sKL 1 sKL+ TM 1

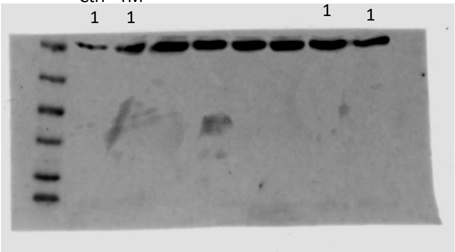

Ctrl 2 TM 2 sKL 2 sK+ TM 2

GRP78

Ctrl 3 TM 3 sKL 3 sK+ TM 3

GRP78

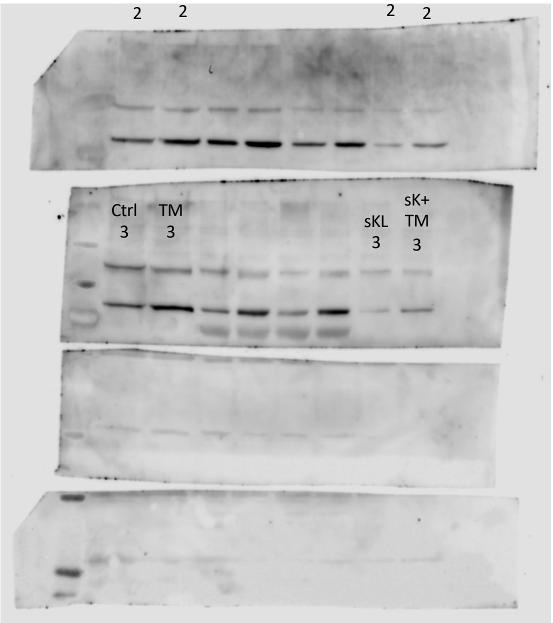

Ctrl 2 TM 2 sKL 2 sK+ TM 2

B-actin

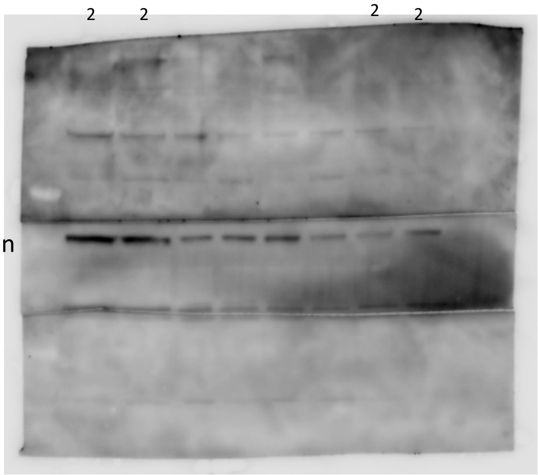

Ctrl 3 TM 3 sKL 3 sK+ TM 3

B-actin

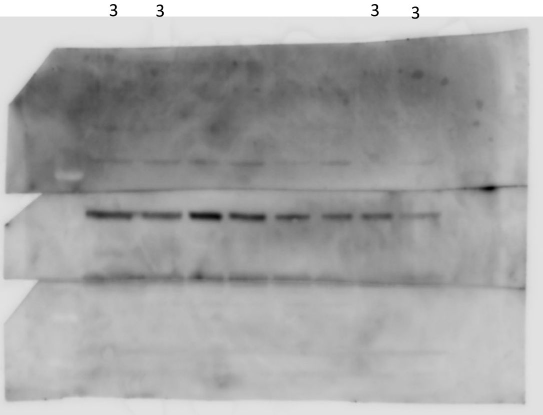

Supplement: Supplementary file 1 — Supplementary information [file 42003_2023_4563_MOESM1_ESM.pdf]
