# Supplementary material for: Soluble Klotho protects against glomerular injury through regulation of ER stress response
Source: Commun Biol. 2023 Feb 22;6:208. doi: 10.1038/s42003-023-04563-1 (PMC9947099; doi:10.1038/s42003-023-04563-1)
Supplement: Supplementary file 4 — Reporting summary [file 42003_2023_4563_MOESM4_ESM.pdf]

nature portfolio

Corresponding author(s): Hannes Olauson

Last updated by author(s): Dec 12, 2022

Reporting Summary

Nature Portfolio wishes to improve the reproducibility of the work that we publish. This form provides structure for consistency and transparency in reporting. For further information on Nature Portfolio policies, see our Editorial Policies and the Editorial Policy Checklist.

Statistics

For all statistical analyses, confirm that the following items are present in the figure legend, table legend, main text, or Methods section.

n/a	Confirmed	
☐	☒	The exact sample size (*n*) for each experimental group/condition, given as a discrete number and unit of measurement
☐	☒	A statement on whether measurements were taken from distinct samples or whether the same sample was measured repeatedly
☐	☒	The statistical test(s) used AND whether they are one- or two-sided<br>*Only common tests should be described solely by name; describe more complex techniques in the Methods section.*
☒	☐	A description of all covariates tested
☒	☐	A description of any assumptions or corrections, such as tests of normality and adjustment for multiple comparisons
☐	☒	A full description of the statistical parameters including central tendency (e.g. means) or other basic estimates (e.g. regression coefficient) AND variation (e.g. standard deviation) or associated estimates of uncertainty (e.g. confidence intervals)
☒	☐	For null hypothesis testing, the test statistic (e.g. *F*, *t*, *r*) with confidence intervals, effect sizes, degrees of freedom and *P* value noted<br>*Give P values as exact values whenever suitable.*
☒	☐	For Bayesian analysis, information on the choice of priors and Markov chain Monte Carlo settings
☒	☐	For hierarchical and complex designs, identification of the appropriate level for tests and full reporting of outcomes
☒	☐	Estimates of effect sizes (e.g. Cohen's *d*, Pearson's *r*), indicating how they were calculated

Our web collection on statistics for biologists contains articles on many of the points above.

Software and code

Policy information about availability of computer code

Data collection	*Provide a description of all commercial, open source and custom code used to collect the data in this study, specifying the version used OR state that no software was used.*
Data analysis	*Provide a description of all commercial, open source and custom code used to analyse the data in this study, specifying the version used OR state that no software was used.*

For manuscripts utilizing custom algorithms or software that are central to the research but not yet described in published literature, software must be made available to editors and reviewers. We strongly encourage code deposition in a community repository (e.g. GitHub). See the Nature Portfolio guidelines for submitting code & software for further information.

Data

Policy information about availability of data

All manuscripts must include a data availability statement. This statement should provide the following information, where applicable:

- Accession codes, unique identifiers, or web links for publicly available datasets
- A description of any restrictions on data availability
- For clinical datasets or third party data, please ensure that the statement adheres to our policy

The data supporting the findings of this study are openly available in repository Gene Expression Omnibus (accession number GSE195641).

Human research participants

Policy information about studies involving human research participants and Sex and Gender in Research.

Reporting on sex and gender	For generation of precision cut kidney slices we used tissue from anonymous nephrectomy patients, for whom we do not know the sex or gender
Population characteristics	For generation of precision cut kidney slices we used tissue from anonymous nephrectomy patients, for whom we do not know the clinical characteristics
Recruitment	For generation of precision cut kidney slices we used tumor free tissue resected by a pathologist and not needed for diagnostic purposes
Ethics oversight	Ethical Review Board in Stockholm, Sweden, archive numbers 2010/579-31 and 2016/615-32

Note that full information on the approval of the study protocol must also be provided in the manuscript.

Field-specific reporting

Please select the one below that is the best fit for your research. If you are not sure, read the appropriate sections before making your selection.

☒ Life sciences ☐ Behavioural & social sciences ☐ Ecological, evolutionary & environmental sciences

For a reference copy of the document with all sections, see nature.com/documents/nr-reporting-summary-flat.pdf

Life sciences study design

All studies must disclose on these points even when the disclosure is negative.

Sample size	Sample sized were calculated using Power calculation to detect a 30% difference in proteinuria (or other outcomes) with an alpha of 0.05 and a power of 80%
Data exclusions	No data were excluded from analysis
Replication	All in vitro and ex vivo experiments were performed in at least three replicates.
Randomization	No randomization was performed. Mice were divided by genotype. For ex vivo studies tissue from the same patients were used for all groups (treatment and control)
Blinding	The researchers were blinded during analysis, and genotypes were revealed during data interpretation

Reporting for specific materials, systems and methods

We require information from authors about some types of materials, experimental systems and methods used in many studies. Here, indicate whether each material, system or method listed is relevant to your study. If you are not sure if a list item applies to your research, read the appropriate section before selecting a response.

Materials & experimental systems

n/a	Involved in the study	
☐	☒	Antibodies
☐	☒	Eukaryotic cell lines
☒	☐	Palaeontology and archaeology
☐	☒	Animals and other organisms
☒	☐	Clinical data
☐	☒	Dual use research of concern

Methods

n/a	Involved in the study	
☒	☐	ChIP-seq
☒	☐	Flow cytometry
☒	☐	MRI-based neuroimaging

Antibodies

Antibodies used	alpha-Klotho clone KM2076, cat#KO603 TransGenics Inc. Lot#TG040121<br>alpha-smooth muscle actin clone 1A4 cat#C6198 Sigma-Aldrich Lot#122897

	β-actin cat#ab8227 Abcam Lot#GR3314266-1<br>Calnexin cat#ab10286 Abcam Lot#GR146087-11<br>GRP78/BiP cat#ADI-SPA-826-D EnzoLife Lot#5021649<br>GRP/BiP clone EPR4041(2) cat#ab108615 Abcam<br>CHOP clone L63F7 cat#2895 Cell Signaling Lot#13<br>Podocin cat#P0372 Sigma-Aldrich Lot#035M4851V<br>Nephrin cat#BP5030 Origene Lot#606051-05<br>GFP cat#ab290 Abcam Lot#GR3270983-1<br>alpha-Tubulin clone DM1A cat#3873S Cell Signaling Lot#15<br>LC3B cat#NB100-2220SS Novus Biologicals Lot#ER<br>Synaptopdin cat#61094 Progen<br>tdTomato cat#TA150129 Origene Lot#R0315<br>Vimentin cat#W5255 Sigma-Aldrich Lot#054M4862V<br>Wilms Tumor-1 cat#ab89901 Abcam Lot#GR3247738-2
Validation	Except for Klotho for which the validation was described throughout the article, all the validations and citations can be found on the manufacturers' websites.

Eukaryotic cell lines

Policy information about cell lines and Sex and Gender in Research

Cell line source(s)	Immortalized mouse podocytes kindly provided by Peter Mundel.
Authentication	The cell line was not authenticated
Mycoplasma contamination	Cells were not tested for mycoplasma
Commonly misidentified lines<br>(See ICLAC register)	*Name any commonly misidentified cell lines used in the study and provide a rationale for their use.*

Animals and other research organisms

Policy information about studies involving animals; ARRIVE guidelines recommended for reporting animal research, and Sex and Gender in Research

Laboratory animals	All the mouse lines (Mus musculus) were on C57Bl6/J background. Mice were used between 7 and 20 weeks of age
Wild animals	No wild animals were used in this study
Reporting on sex	The incidence of chronic kidney disease is usually greater in women compared with age-matched men. Nevertheless, kidney function declines faster in men than women and mortality is higher among men at all stages of CKD. Experimental evidence suggests that both estrogens and androgens play an important role in the pathophysiology of renal disease. However, causes for these gender differences are not really known. For that reason, patient and mouse selection for the study was done without any sex bias.
Field-collected samples	This study did not involve samples collected from the field.
Ethics oversight	The ethical approval for this study was delivered by the Swedish Board of Agriculture (Jordbruksverket) under DNR1336-19 and DNR41-15

Note that full information on the approval of the study protocol must also be provided in the manuscript.

Dual use research of concern

Policy information about dual use research of concern

Hazards

Could the accidental, deliberate or reckless misuse of agents or technologies generated in the work, or the application of information presented in the manuscript, pose a threat to:

No	Yes	
☒	☐	Public health
☒	☐	National security
☒	☐	Crops and/or livestock
☒	☐	Ecosystems
☒	☐	Any other significant area

Experiments of concern

Does the work involve any of these experiments of concern:

No	Yes	
☒	☐	Demonstrate how to render a vaccine ineffective
☒	☐	Confer resistance to therapeutically useful antibiotics or antiviral agents
☒	☐	Enhance the virulence of a pathogen or render a nonpathogen virulent
☒	☐	Increase transmissibility of a pathogen
☒	☐	Alter the host range of a pathogen
☒	☐	Enable evasion of diagnostic/detection modalities
☒	☐	Enable the weaponization of a biological agent or toxin
☒	☐	Any other potentially harmful combination of experiments and agents
